# The genetic basis for adaptation of model-designed syntrophic co-cultures

**DOI:** 10.1371/journal.pcbi.1006213

**Published:** 2019-03-01

**Authors:** Colton J. Lloyd, Zachary A. King, Troy E. Sandberg, Ying Hefner, Connor A. Olson, Patrick V. Phaneuf, Edward J. O’Brien, Jon G. Sanders, Rodolfo A. Salido, Karenina Sanders, Caitriona Brennan, Gregory Humphrey, Rob Knight, Adam M. Feist

**Affiliations:** 1 Department of Bioengineering, University of California, San Diego, La Jolla, United States of America; 2 Bioinformatics and Systems Biology Program, University of California, San Diego, La Jolla, United States of America; 3 Department of Pediatrics, University of California, San Diego, La Jolla, United States of America; 4 Cornell Institute of Host-Microbe Interactions and Disease, Cornell University, Ithaca, United States of America; 5 Center for Microbiome Innovation, University of California, San Diego, La Jolla, United States of America; 6 Department of Computer Science and Engineering, University of California, San Diego, La Jolla, United States of America; 7 Novo Nordisk Foundation Center for Biosustainability, Technical University of Denmark, Denmark; Ecole Polytechnique Fédérale de Lausanne, SWITZERLAND

## Abstract

Understanding the fundamental characteristics of microbial communities could have far reaching implications for human health and applied biotechnology. Despite this, much is still unknown regarding the genetic basis and evolutionary strategies underlying the formation of viable synthetic communities. By pairing auxotrophic mutants in co-culture, it has been demonstrated that viable nascent *E*. *coli* communities can be established where the mutant strains are metabolically coupled. A novel algorithm, OptAux, was constructed to design 61 unique multi-knockout *E*. *coli* auxotrophic strains that require significant metabolite uptake to grow. These predicted knockouts included a diverse set of novel non-specific auxotrophs that result from inhibition of major biosynthetic subsystems. Three OptAux predicted non-specific auxotrophic strains—with diverse metabolic deficiencies—were co-cultured with an L-histidine auxotroph and optimized via adaptive laboratory evolution (ALE). Time-course sequencing revealed the genetic changes employed by each strain to achieve higher community growth rates and provided insight into mechanisms for adapting to the syntrophic niche. A community model of metabolism and gene expression was utilized to predict the relative community composition and fundamental characteristics of the evolved communities. This work presents new insight into the genetic strategies underlying viable nascent community formation and a cutting-edge computational method to elucidate metabolic changes that empower the creation of cooperative communities.

## Author summary

Many basic characteristics underlying the establishment of cooperative growth in bacterial communities have not been studied in detail. The presented work sought to understand the adaptation of syntrophic communities by first employing a new computational method to generate a comprehensive catalog of *E*. *coli* auxotrophic mutants. Many of the knockouts in the catalog had the predicted effect of disabling a major biosynthetic process. As a result, these strains were predicted to be capable of growing when supplemented with many different individual metabolites (i.e., a non-specific auxotroph), but the strains would require a high amount of metabolic cooperation to grow in community. Three such non-specific auxotroph mutants from this catalog were co-cultured with a proven auxotrophic partner *in vivo* and evolved via adaptive laboratory evolution. In order to successfully grow, each strain in co-culture had to evolve under a pressure to grow cooperatively in its new niche. The non-specific auxotrophs further had to adapt to significant homeostatic changes in cell’s metabolic state caused by knockouts in metabolic genes. The genomes of the successfully growing communities were sequenced, thus providing unique insights into the genetic changes accompanying the formation and optimization of the viable communities. A computational model was further developed to predict how finite protein availability, a fundamental constraint on cell metabolism, could impact the composition of the community (i.e., the relative abundances of each community member).

## Introduction

Microbial communities are capable of accomplishing many intricate biological feats due to their ability to partition metabolic functions among community members. Therefore, these microbial consortia have the attractive potential to accomplish complex tasks more efficiently than a single wild-type or engineered microbial strain. Past applications include applying communities to aid in waste decomposition, fuel cell development, and the creation of biosensors [1]. In the field of metabolic engineering, microbial communities have now been engineered capable of enhancing product yield or improving process stability by partitioning catalytic functions among community members [2–8]. Beyond biotechnology applications, studying microbial communities also has important health implications. This includes providing a better understanding of the gut microbiome and how it is affected by diet and other factors [9,10]. For example, metabolic cross-feeding in communities has been shown to have a role in modulating the efficacy of antibiotics treatments [11]. New computational and experimental approaches to better understand the characteristics of viable microbial communities could therefore have far reaching implications.

Synthetic communities have been constructed to study their interactions and new metabolic capabilities. One such study encouraged synthetic symbiosis between *E*. *coli* strains by co-culturing an L-isoleucine auxotroph with a L-leucine auxotroph [12,13]. It was observed that the community was able to grow in glucose minimal media without amino acid supplementation due to amino acid cross-feeding between the mutant pairs. Mee *et al*. expanded upon this work by studying all possible binary pairs of 14 amino acid auxotrophs and developing methods to predict the results of combining the auxotrophic strains into 3-member, 13-member, and 14-member communities [14]. Similarly, Wintermute *et al*. observed community formation using a more diverse set of auxotrophs by co-culturing 46 conditionally lethal single gene knockouts from the *E*. *coli* Keio collection [15]. This work demonstrated that synthetic mutualism was possible in strains beyond amino acid auxotrophs [16]. These studies also demonstrated that new viable communities can be established in relatively short time frames (<4 days) by pairing auxotrophic strains.

In addition to establishing syntrophic growth, nascent auxotrophic communities can be optimized by adaptive laboratory evolution (ALE) [17]. Expanding upon the experimental work in Mee *et al*. [14], Zhang *et al*. performed ALE on one of the co-culture pairs: a L-lysine auxotroph paired with a L-leucine auxotroph [17]. Separate co-cultures evolved to growth rates 3-fold greater than the parent, which was accomplished, in part, by forming different auxotroph strain abundances within the community. Similarly, Marchal *et al*. evolved co-cultures of two *E*. *coli* amino acid auxotrophs and sequenced the endpoint strains. This data was leveraged to identify mutations hinting at changes in the spatial structure that occurred during the evolution [18]. Studies of evolved co-culture pairs composed of different microbial species have also used sequencing data and mutational analysis as a crucial component of interpreting adaptive strategies [19,20]. The success of the above work demonstrated that ALE can be used to optimize auxotrophic communities and that mutational data provide valuable insight into mechanisms underlying the evolved improvements in community growth rates.

Computational methods have been established to study the characteristics of microbial communities. These methods often apply genome-scale metabolic models (M-models) [21–23]. Computational models have been created that use multicompartmental flux balance analysis (FBA) [23–26], dynamic flux balance analysis (dFBA) [17,27], dFBA integrated with spatial diffusion of extracellular metabolites (COMETS) [28], and FBA with game theory [29]. Novel algorithms have also been developed to describe general community characteristics (OptCom [30]) and dynamics (d-OptCom [31]). These algorithms employ a bilevel linear programming problem to find the metabolic state that maximizes community biomass while also maximizing the biomass objectives of each individual species [32]. Numerous ecological models have also been formulated to describe community dynamics [33–35].

Despite the significant advances made by the above modeling approaches, most methods were not intended to model suspension batch ALE experiments. For instance, ALE batch experiments in suspension assume growth in excess, well-mixed nutrients, thus negating the need for diffusion considerations (COMETS) or dynamic shifts in nutrient concentrations (dFBA). Also, in order for the strains to persist serial passage in an ALE experiment, it can be assumed that the cells in co-culture are growing, on average, at the same rate, thus negating the need for a bilevel growth objective that allows for varying growth rates of community members (OptCom). Additionally, given the growing appreciation for the role limited protein availability has on governing fundamental bacterial growth characteristics [36], it is likely that protein allocation plays a role in defining fundamental community characteristics as well. Therefore, there is a need for an applicable approach to model this experimental condition in a way that accounts for the protein cost of metabolism.

**Fig 1:**
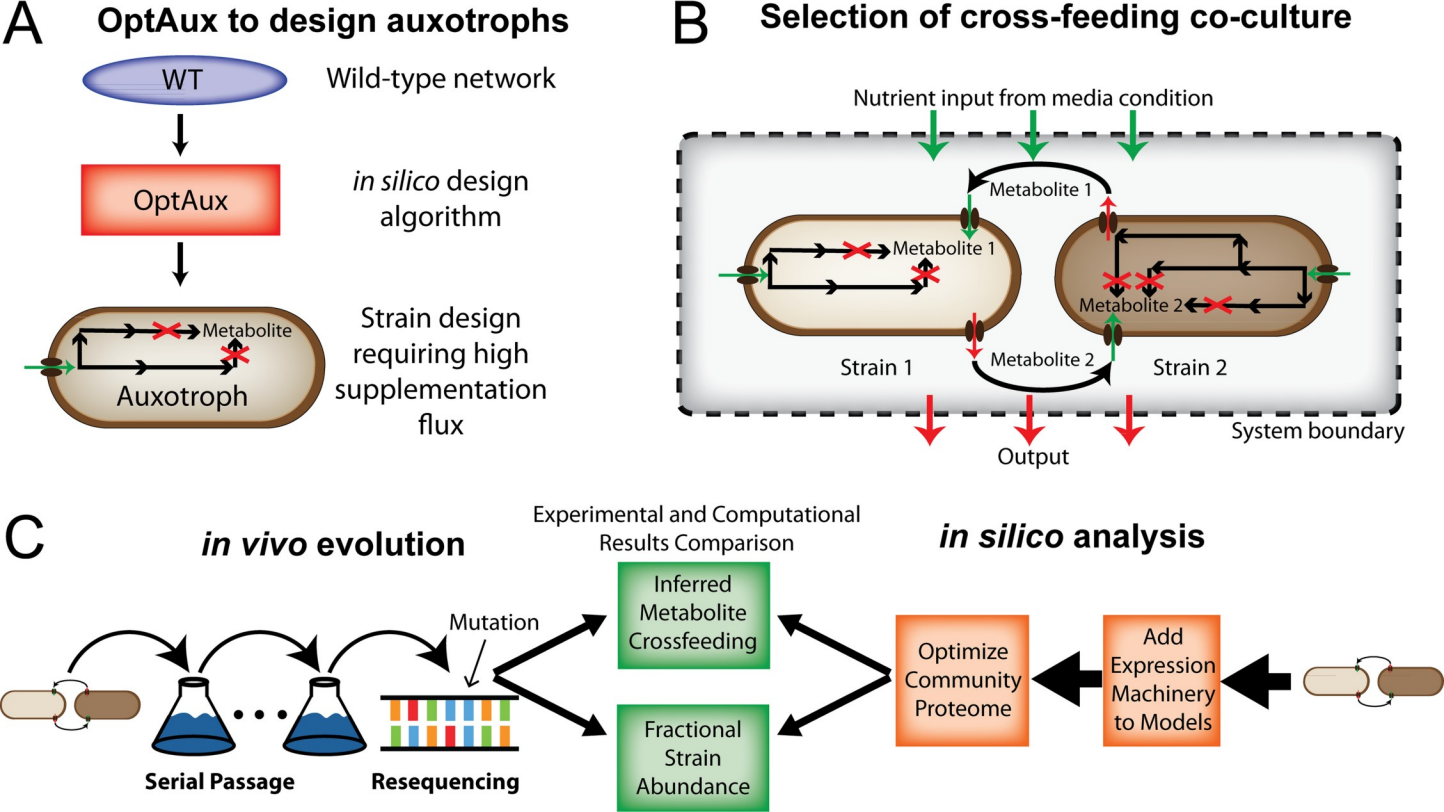
Study overview. (**A**) An algorithm was developed to *de novo* predict reaction deletions that will produce *E*. *coli* strains auxotrophic for a target metabolite. (**B**) From the set of auxotrophic strain designs, pairs were selected to determine whether they were capable of forming a viable syntrophic community. (**C**) The chosen co-cultures were both evolved via adaptive laboratory evolution and modeled using a genome-scale model of *E*. *coli* metabolism and expression (ME-model) [39,44]. The model predictions of fractional strain abundances and metabolite cross-feeding were compared to inferred results from the co-culture evolution experiments.

Here, we elucidate the genetic mechanisms underlying the formation of syntrophy between co-cultures of auxotrophic mutants containing diverse biosynthetic deficiencies. We first introduce the OptAux algorithm for designing auxotrophic strains that require high amounts of supplemented metabolites to grow (**Fig 1A**). The OptAux solutions provided a catalog of auxotrophic mutants representing a diverse set of metabolic deficiencies. From the catalog, four auxotrophic mutants were selected to co-culture and optimized via adaptive laboratory evolution (ALE) (**Fig 1B**). To increase the growth rate of the nascent co-culture communities, significant metabolic rewiring had to occur to allow the strains to cross-feed the high levels of the necessary metabolites. Some strains additionally had to adapt to marked changes in their homeostatic metabolic state, resulting from the inhibition of a major biosynthetic subsystem. The genetic basis accompanying this rewiring was assessed by analyzing the genetic changes (mutations and observed genome region duplications) over the course of the ALE. This mutational analysis further enabled predictions of primary metabolite cross-feeding and community composition.

To study the characteristics of the ALE-optimized communities, a community model of metabolism and expression (ME-model) was constructed [37–39] (**Fig 1C**). Such a modeling approach was necessary since previous methods of genome-scale community modeling have focused on studying the metabolic flux throughout community members (using M-models) without consideration of the enzymatic cost of the proteins that drive these metabolic processes. As proteome optimization via niche partitioning and cell specialization is a driving factor of viable community formation in ecological systems [40–43], it is essential to consider proteomic constraints when studying bacterial communities. To this end, community ME-models were utilized to interpret the nascent communities.

## Results

### OptAux development and simulation

**Fig 2:**
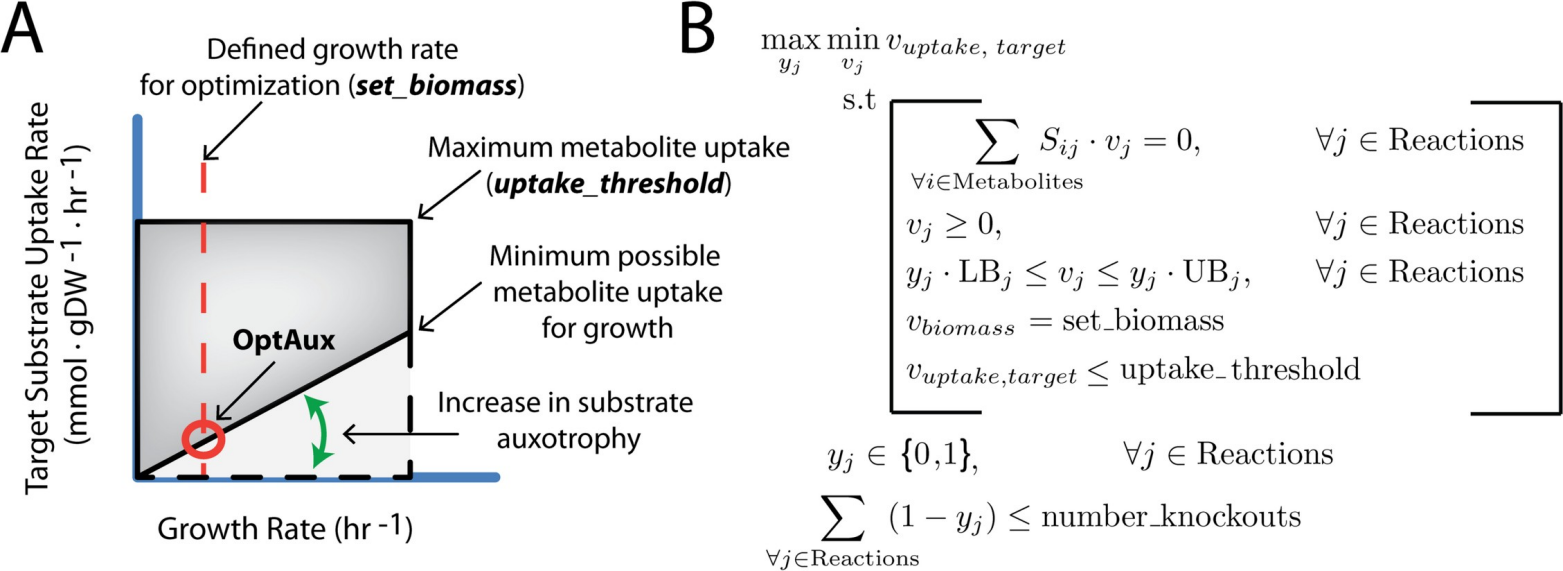
OptAux design. (**A**) OptAux was developed to maximize the minimum possible uptake of a target metabolite required for the model to grow. In other words, OptAux tries to increase the flux value at the intersection of the defined growth rate (*set_biomass*) and the minimum possible metabolite uptake flux (depicted with the red circle). Unlike algorithms such as OptKnock with tilting [47] and RobustKnock [48], the OptAux optimization occurs at a predetermined growth rate as opposed to imposing an inner growth rate optimization. This change was made to ensure that all OptAux designs will computationally require the uptake of a target metabolite at all growth rates, particularly low growth rates. The dotted lines show the required uptake for the metabolite with no genetic interventions. In this case, uptake of the target metabolite is not required at any growth rate. The solid black lines depicts the maximum and minimum uptake required for a particular metabolite in an OptAux designed strain. (**B**) The OptAux optimization problem. See **Methods** for further description of the algorithm and underlying logic.

The OptAux algorithm was designed to find metabolic reactions in *E*. *coli* that, when knocked out, will result in novel auxotrophies. This algorithm was implemented by selecting a metabolite of interest and applying OptAux to identify sets of reaction knockouts that will increase the uptake of the metabolite required for the cell to computationally grow (**Fig 2A**). OptAux was built by modifying an existing concept introduced for designing metabolite producing strains [45] which was later additionally implemented in a mixed-integer linear programming (MILP) algorithm (RobustKnock [46]). Three key modifications were made to derive OptAux from RobustKnock. **First,** the inner growth rate optimization was removed so that OptAux can be run at a predetermined growth rate (*set_biomass* constraint, **Fig 2B**). This ensures that OptAux designs computationally require the uptake of the target metabolite at all growth rates (**Fig 2A, Figure A in S1 Appendix**). **Second,** the objective coefficient was reversed in order to allow the algorithm to optimize for metabolite uptake as opposed to secretion. **Third**, a constraint was added to allow adjustments in the “specificity” of OptAux solutions (see **Methods**). This constraint allows the OptAux simulation to uptake any additional metabolite that can be consumed by the model (*competing_metabolite_uptake_threshold* constraint, **Fig 2B**). Without this constraint, many OptAux predicted designs have the potential to also grow in the presence of metabolites other than the target metabolite. For instance, it is possible that OptAux-predicted L-glutamate auxotroph mutants could alternatively grow when supplemented with L-glutamine or other metabolites as well. Therefore, “specificity”, in this case, refers to whether the mutant strain will be auxotrophic for a given metabolite in the presence of other metabolites. High specificity solutions are auxotrophic for only one metabolite, regardless of whether other metabolites are present. The implementation described above allowed OptAux to identify strain designs requiring the targeted metabolite at all growth rates with varying degrees of metabolite specificity.

OptAux was utilized on the *i*JO1366 M-model of *E*. *coli* K-12 MG1655 [49,50] to comprehensively examine auxotrophic strain designs. OptAux was run with 1, 2, and 3 reaction knockouts for 285 metabolite uptake reactions using 4 different *competing_metabolite_uptake_threshold* values (**S1 Data**). Of the given solutions, 233 knockout sets were found to be capable of producing 61 unique strain auxotrophies. This set of strain designs provides an expansive look into the auxotrophies possible in the *E*. *coli* K-12 MG1655 metabolic network, which could be used to understand the possible niches that *E*. *coli* could inhabit in natural or synthetic communities [51].

#### OptAux solution characteristics

**Fig 3:**
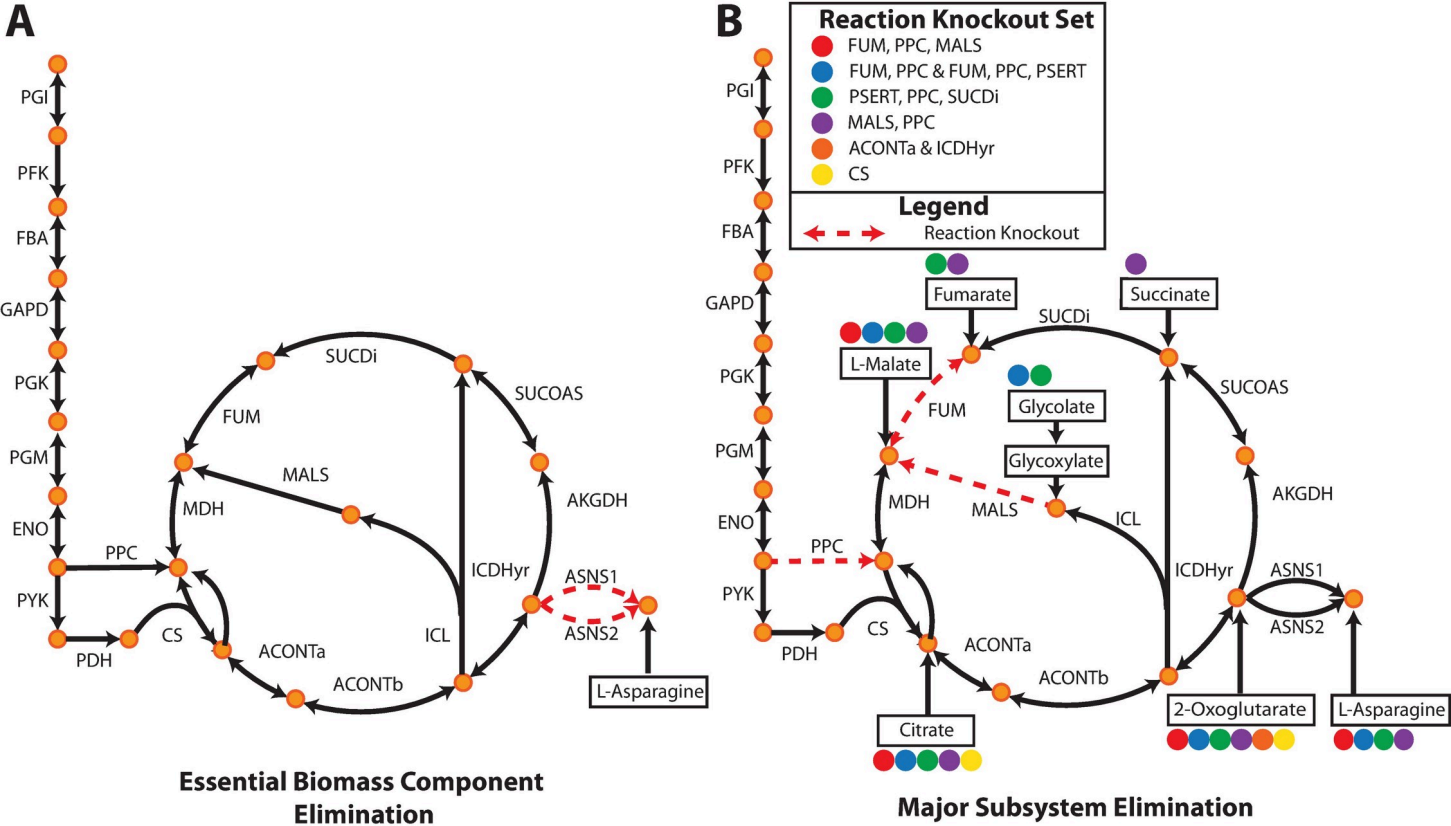
OptAux solutions. Two major solution types are possible depending on the parameters used when running OptAux. (**A**) **Essential Biomass Component Elimination** designs, like the ASNS1 and ASNS2 knockout shown, can grow only when one specific metabolite is supplemented. For the case shown, this metabolite is L-asparagine. (**B**) Alternatively, **Major Subsystem Elimination** designs have a set of alternative metabolites that can individually restore growth in these strains. Examples of these designs are shown for citric acid cycle knockouts sets. One specific three reaction knockout design (FUM, PPC, MALS) is shown in red dashed lines where four metabolites in the figure can individually rescue this auxotroph (marked with solid red circles). The metabolites that can restore growth for each of the knockout strain designs listed in the legend are indicated by the colored circles.

The OptAux strain designs were broken into two major categories based on the number of individual metabolites that, when supplemented, can restore cell growth: **1) Essential Biomass Component Elimination Designs (EBC, Fig 3A)** and **2) Major Subsystem Elimination Designs (MSE, Fig 3B)**. The EBC designs are characterized as auxotrophic strains with high metabolite specificity. They were broken into two subcategories: specific auxotrophs (only one metabolite can restore growth, **Figure B in S1 Appendix**) which consisted of 107 (20 unique) knockout sets and semi-specific auxotrophs (defined as strains in which less than 5 metabolites individually can restore growth, **Figure B in S1 Appendix**) which consisted of 67 (21 unique) knockout sets. The specific and semi-specific EBC designs were preferred at high *competing_metabolite_uptake_threshold* values.

There is notable overlap between OptAux predicted EBC designs (or those that are computationally identical), and known *E*. *coli* auxotrophic mutants [14,52–63]. A summary of experimentally characterized OptAux designs is presented in **Table A in S1 Appendix**. Of note, there are 4 designs that were not found to be previously characterized in the scientific literature, and these present potential novel *E*. *coli* auxotrophs.

MSE designs were preferred at low *competing_metabolite_uptake_threshold* values and produce *E*. *coli* mutant strains with a diverse set of major metabolic deficiencies. These designs were defined as highly non-specific auxotrophic strains in which 5 or more metabolites could individually restore growth in the mutant strain. MSE designs consisted of the remaining 59 (20 unique) sets of knockouts. The MSE knockout strategy was often accomplished through knockouts that block metabolic entry points into key biosynthetic subsystems (**Figure B in S1 Appendix**). One such example of an MSE design is given in **Fig 3B**. Here a three reaction knockout design of the FUM, PPC, and MALS reactions can be rescued by one of the four compounds in the figure (i.e., citrate, L-malate, 2-oxoglutarate, or L-asparagine) at an average required uptake flux of 0.40 mmol gDW ^-1^ hr ^-1^ to grow at a rate of 0.1 hr ^-1^. These rates are higher than the fluxes needed to rescue the EBC design in **Fig 3A**, which requires L-asparagine uptake of 0.024 mmol gDW ^-1^ hr ^-1^ at a rate of 0.1 hr ^-1^. Another example of a novel MSE design was a glutamate synthase (GLUSy) and glutamate dehydrogenase (GLUDy) double knockout which effectively blocks the entry of nitrogen into amino acid biosynthesis by preventing its incorporation into 2-oxoglutarate to produce L-glutamate. This renders the cell unable to produce all amino acids, nucleotides, and several cofactors. In order to grow at a rate of 0.1 hr ^-1^, this strain is computationally predicted to require one of 19 individual metabolites at an average uptake of 0.62 mmol gDW ^-1^ hr ^-1^ (**S2 Data**).

MSE designs are of particular interest as they are often unique, non-trivial, and have not been studied in the context of *E*. *coli* auxotrophies. However, some of the MSE single knockouts have been used for a large-scale study of auxotrophic co-culture short term growth [16]. Since these predicted MSE knockouts disrupt major metabolic flows in the cell’s biochemical network, they produce auxotrophies that require much larger amounts of metabolite supplementation to grow, compared to EBC designs (e.g., **Figure C in S1 Appendix**). To grow in co-culture, MSE *E*. *coli* mutants would require a pronounced metabolic rewiring and likely additional adaptation to a new homeostatic metabolic state, making them attractive to study from a microbial community perspective. Additionally, any strain paired with an MSE strain in co-culture would be required to provide a relatively high amount of the MSE strain’s auxotrophic metabolites to enable community growth.

### Adaptive laboratory evolution of auxotrophic *E*. *coli* co-cultures

To demonstrate how the OptAux algorithm can be leveraged to design strains and co-culture communities, *E*. *coli* auxotrophic mutants were validated in the wet lab and evolved in co-culture. Three communities were tested, each consisting of pairwise combinations of four OptAux predicted auxotrophs. This included one EBC design, Δ*hisD*, which was validated as an L-histidine auxotroph, paired with each of three MSE designs, Δ*pyrC*, Δ*gltA*Δ*prpC*, and Δ*gdhA*Δ*gltB*. These three MSE strains had diverse metabolic deficiencies, including disruptions in pyrimidine synthesis, TCA cycle activity, and nitrogen assimilation into amino acids, respectively (**Table B in S1 Appendix**). The Δ*pyrC* mutant was computationally predicted to be capable of growing when supplemented with one of 20 metabolites in *i*JO1366, and the Δ*gltA*Δ*prpC* and Δ*gdhA*Δ*gltB* mutants were predicted to grow in the presence of 14 and 19 metabolites, respectively (**S2 Data, Table D in S1 Appendix**).

**Fig 4:**
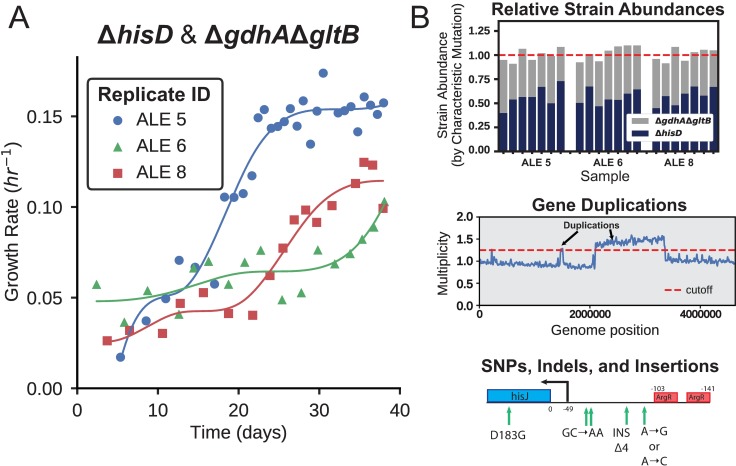
Representative example of an adaptive laboratory evolution and its downstream analysis. (**A**) *E*. *coli* co-cultures were evolved over a 40 day period and the growth rate was periodically measured. Over this time period the co-cultures evolved the capability to establish syntrophic growth, indicated by the improvement in community growth rate. (**B**) Each of the sampled co-cultures were sequenced at multiple points during the evolution. This information was used to predict the fractional strain abundances of each of the co-culture members (top panel, bars represent the computed fractional abundance of the strains in the legend). Sequencing data was also used to identify duplications in genome regions of the community members (middle panel) and infer causal mutations that improved community fitness (bottom panel). The complete set of ALE growth trajectories, inferred strain abundances, gene region duplications, and mutational analysis can be found in **S1 Appendix, S3 Data, S4 Data,** and **Figs 5–7**.

Four replicates of each co-culture were inoculated and initially exhibited low growth rates (< 0.1 hr ^-1^), suggesting the strains initially showed minimal cooperativity or metabolic cross-feeding (**Figure D in S1 Appendix**). Following approximately 40 days of ALE, all 3 co-culture combinations had evolved to establish a viable syntrophic community, indicated by an increase in the co-culture growth rate. There was diversity in the endpoint batch growth rates among the independently evolved triplicates for each of the Δ*hisD* & Δ*pyrC* and the Δ*hisD* & Δ*gdhA*Δ*gltB* co-cultures, with endpoint growth rates ranging from 0.09–0.15 hr ^-1^ and 0.08–0.15 hr ^-1^, respectively. The four successfully evolved independent replicates for the Δ*hisD* & Δ*gltA*Δ*prpC* co-cultures also showed endpoint growth rate diversity ranging from 0.12–0.19 hr ^-1^ (**Table 1, Fig 4A).** The relatively large range in endpoint growth rates for all co-cultures could suggest that a subset of replicates evolved to a less optimal state and thus could potentially be further improved if given more time to evolve. Alternatively, the slower growing co-cultures could have found a genetic state that resulted in a local maxima, rendering the co-culture less likely to increase its growth rate further.

**Table 1:** Starting and final growth rates, along with fractional strain abundance of the *Δhi*sD strain (by characteristic mutation), for each ALE lineage. The cumulative number of cell division events that occurred throughout the experimental evolutions are also provided [64].

Combo	ALE #	Starting growth rate (hr^-1^)	Final growth rate (hr^-1^)	Relative Abundance of Δ*hisD* (by Characteristic Mutation)	Cumulative Cell Divisions (x 10^11^)
**Δ*hisD* & Δ*pyrC***	2	0.03 ± 0.01	0.09 ± 0.02	0.29 ± 0.06	4.63
	3		0.15 ± 0.01	0.25 ± 0.09	3.79
	4		0.10 ± 0.02	0.21 ± 0.10	4.58
**Δ*hisD* & Δ*gdhA*Δ*gltB***	5	0.04 ± 0.02	0.15 ± 0.01	0.57 ± 0.09	6.06
	6		0.08 ± 0.01	0.55 ± 0.06	3.46
	8		0.10 ± 0.02	0.57 ± 0.09	3.04
**Δ*hisD* & Δ*gltA*Δ*prpC***	9	0.09 ± 0.02	0.19 ± 0.01	0.60 ± 0.10	7.50
	10		0.12 ± 0.02	0.50 ± 0.06	2.88
	11		0.13 ± 0.01	0.57 ± 0.09	4.77
	12		0.19 ± 0.01	0.56 ± 0.05	3.57

To probe the adaptive strategies of the three co-culture pairs, the genomes of the populations were sequenced at several time points over the course of the 40 day evolution (**Fig 4A**). The sequencing data was used to identify genome region duplications and acquired mutations (**Fig 4B**), providing insight into the specific mechanisms employed by the co-cultures to establish cooperation.

The relative strain abundance of each mutant was tracked to observe the community composition throughout the course of the evolution. Each starting strain contained at least one unique characteristic mutation (**Table C in S1 Appendix**) that could act as a barcode to track the community composition (**Fig 4B, Table 1**). The breseq mutation identification software [65] was used to report the frequency of each of these characteristic mutations within a sequenced co-culture. The characteristic mutation frequency was then used to approximate the fraction of each strain within the co-culture population. This analysis showed that 2 of the 3 co-culture combinations maintained similar relative fractions of the two member strains, whereas one co-culture, Δ*hisD* & Δ*pyrC*, consistently maintained a relative Δ*pyrC* abundance of around three quarters of the total population (71–79%, **Table 1**). The strain’s prevalence in the community could potentially be overestimated if the strain’s characteristic mutations fell within duplicated genome regions. To account for this possibility, the relative abundance of each strain in the populations was additionally computed by comparing the read coverage of the knocked out genes for each mutant relative to the average read depth. This orthogonal method gave predictions consistent with those obtained using the characteristic mutation-based method (**Figures E-F in S1 Appendix**).

Following the evolutions it was confirmed that all collected ALE endpoint clones remained auxotrophic and had not evolved the ability to grow in glucose M9 minimal media. Given that only the large subunit (*gltB*) of glutamate synthase (catalyzes both glutamate synthase and glutamate dehydrogenase reactions, **Table B in S1 Appendix**) was knocked out, it was important to verify that the cell could not adapt to restore glutamate synthase functionality using only the small subunit (*gltD*) [66].

#### Mutations targeting metabolite uptake/secretion

**Fig 5:**
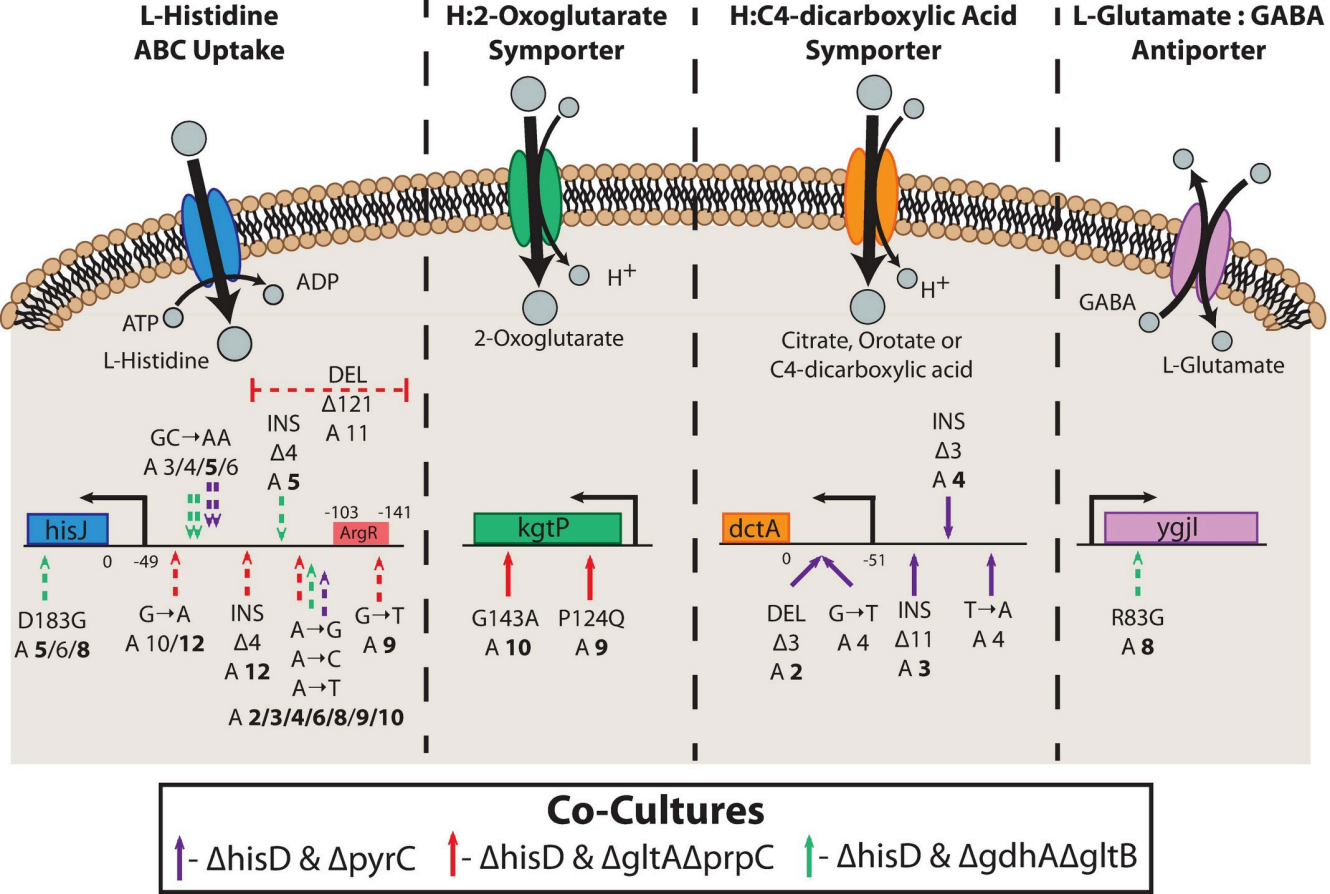
Mutations affecting inner membrane metabolite transport. Mutations were observed that possibly affect the activity of four inner membrane transporters. A schematic of the function or putative function of each transporter is shown. Depicted below the schematics are the locations of the observed mutations on the operon encoding each of the enzymatic complexes. For example, all ten evolved Δ*hisD* strain endpoints possessed at least one mutation in or upstream of *hisJ*. This operon includes genes coding for HisJMPQ, the four subunits of an L-histidine ABC uptake system. A depiction of the activity of this complex is shown, in which energy from ATP hydrolysis is used to transport L-histidine into the cytosol from the periplasm. Mutations are indicated on the operon schematics if mutations appear at >10% frequency in more than one flask in an ALE lineage, and ALE numbers are in bold if the mutation appears in the endpoint clone. The mutations indicated with a dashed arrow occured in the Δ*hisD* strain and a solid arrow indicates they occured in Δ*hisD* strain’s partner MSE strain.

Several evolutionary strategies were observed in the mutations identified across the ten successfully evolved co-culture lineages (**Tables E-G in S1 Appendix**). One ubiquitous strategy across all three co-culture pairs, however, was to acquire mutations within or upstream of inner membrane transporter genes. For instance, numerous mutations were observed in every co-culture lineage in the *hisJ* ORF or upstream of the operon containing *hisJ*. This operon contains all four genes (*hisJ*, *hisM*, *hisP*, *hisQ*) composing the L-histidine ABC uptake complex, the primary mechanism for L-histidine uptake in *E*. *coli* K-12 MG1655 [67]. Seven mutations were found in the region directly upstream of the operon’s transcription start site (**Fig 5**). Two of the seven mutations were further observed in more than one co-culture pairing, with a SNP in one position (A->G, A->C, or A->T at 86 base pairs upstream of *hisJ*) appearing to be particularly beneficial as it was identified in the endpoint clone of every lineage except one (ALE #5). In three ALEs, a mutation was observed within the *hisJ* ORF that resulted in a substitution of the L-aspartate residue at the 183 position by glycine. Based on the protein structure, this substitution could disrupt two hydrogen bond interactions with the bound L-histidine ligand in the periplasm [68]. Alternatively, this mutation could function to modulate translation of the *hisJ* operon by altering its mRNA secondary structure. Further mutations were observed that could affect the binding of the ArgR repressor upstream of the *hisJMPQ* operon (**Table E in S1 Appendix**) or affect the activity of the ArgR protein itself (**Table F in S1 Appendix**). This included a 121 base pair deletion and a SNP in the ArgR repressor binding site upstream of *hisJ* (**Fig 5**). The mutation in the *argR* ORF consisted of a frameshift insertion early in the coding sequence and persisted throughout ALE #8, appearing in the *ΔhisD* endpoint clone (**Table F in S1 Appendix**). ArgR functions to repress L-arginine uptake and biosynthesis as well as repress the L-histidine ABC uptake complex [69] in response to elevated L-arginine concentrations. All of the above mutations could improve L-histidine uptake in the Δ*hisD* strains either by increasing the expression, improving the efficacy, or preventing ArgR mediated repression of the HisJMPQ ABC uptake system.

Beyond improving the uptake of L-histidine in the Δ*hisD* strain, mutations were observed that could improve metabolite uptake in the partnering strain. For instance, in the Δ*hisD* & Δ*gltA*Δ*prpC* co-culture, two of the evolutions acquired mutations in the *kgtP* ORF (a transporter of 2-oxoglutarate [70]) that were also present in the Δ*gltA*Δ*prpC* endpoint clones. These mutations include a substitution of an L-proline residue with an L-glutamine at the 124 position and a substitution of a glycine residue with an L-alanine at the 143 position (**Table E in S1 Appendix**). These two substitutions occurred in the fourth transmembrane helix in the protein and a cytoplasmic region [71], respectively. These mutations could act to augment the activity of the transporter or modulate its expression by changing the mRNA secondary structure. The mutations further could complement the characteristic mutation upstream of the *kgtP* ORF observed in the starting clone of the Δ*gltA*Δ*prpC* mutant (**Table C in S1 Appendix**). Both the accumulation of mutations associated with this transporter and the fact that the citrate synthase knockout mutant is computationally predicted to grow in the presence of 2-oxoglutarate suggest that Δ*gltA*Δ*prpC* could be cross-fed 2-oxoglutarate *in vivo* when in co-culture (**Table 2**).

**Table 2:** Metabolite being cross-fed by the Δ*hisD* strain to its partner strain, as inferred from sequencing data.

Pair with Δ*hisD*	Inferred Metabolite	Mutation Evidence	Duplication Evidence
**Δ*pyrC***	Orotate	Mutations upstream of *dctA* in Δ*pyrC* strain in all ALEs (**Fig 5**)	Broad duplication in portion of genome containing *dctA* coding region in all ALEs (**Figure J in S1 Appendix, S4 Data**)
**Δ*gdhA*Δ*gltB***	L-Glutamate	Ale #8 mutation in *ygjI* ORF in Δ*hisD* strain (**Fig 5**)	ALE #5/6 targeted duplications in gltJ coding region (**Fig 7**, **Figure I in S1 Appendix**)
			ALE #5 transient duplication in abgT coding region (**Fig 7**)
**Δ*gltA*Δ*prpC***	2-Oxoglutarate	Starting mutation upstream of *kgtP* in Δ*gltA*Δ*prpC* strain (**Table E in S1 Appendix**)	-
		ALE #9/10 mutations in *kgtP* ORF in Δ*gltA*Δ*prpC* strain (**Fig 5**)	

For the *ΔhisD* & *ΔpyrC* co-culture, mutations were consistently observed upstream of *dctA* that could function to better facilitate the uptake of a metabolite being cross-fed from the Δ*hisD* strain to the Δ*pyrC* strain. The three independently evolved lineages each acquired at least one mutation upstream of *dctA*, which were confirmed to be in all Δ*pyrC* endpoint clones (**Table G in S1 Appendix**). The gene product of *dctA* functions as a proton symporter that can uptake orotate, malate, citrate, and C4-dicarboxylic acids [72] (**Fig 5**). Model simulations of a Δ*pyrC* strain predicted that growth is possible with orotate supplementation, but not with any of the other metabolites known to be transported by the *dctA* gene product. Thus, it is possible these mutations could act to increase the activity of this transporter to allow the *ΔpyrC* strain to more efficiently uptake the orotate being cross-fed by the *ΔhisD* strain (**Table 2**).

Lastly, one lineage of the Δ*hisD* & Δ*gdhA*Δ*gltB* co-culture acquired a SNP in the *ygjI* coding region and was present in the Δ*hisD* endpoint clone. This SNP resulted in a substitution of L-arginine for glycine at position 83, (**Table F in S1 Appendix**) within a periplasmic region and one residue prior to a transmembrane helix of the protein [73]. The function of this protein has not been experimentally confirmed, but based on sequence similarity, it is predicted to be a GABA:L-glutamate antiporter [74]. Given that this mutation was seen in the *ΔhisD* clone, it is possible that this mutation had the effect of increasing the strain’s secretion of 4-aminobutyrate (GABA) or L-glutamate by increasing the expression or modulating the activity of YgjI. Such a mutation could improve the community growth rate by facilitating the cross-feeding of either these metabolites to the Δ*gdhA*Δ*gltB* strain since this strain is predicted to grow when supplemented with either GABA or L-glutamate (**Table D in S1 Appendix**).

#### Mutations targeting nitrogen regulation

**Fig 6:**
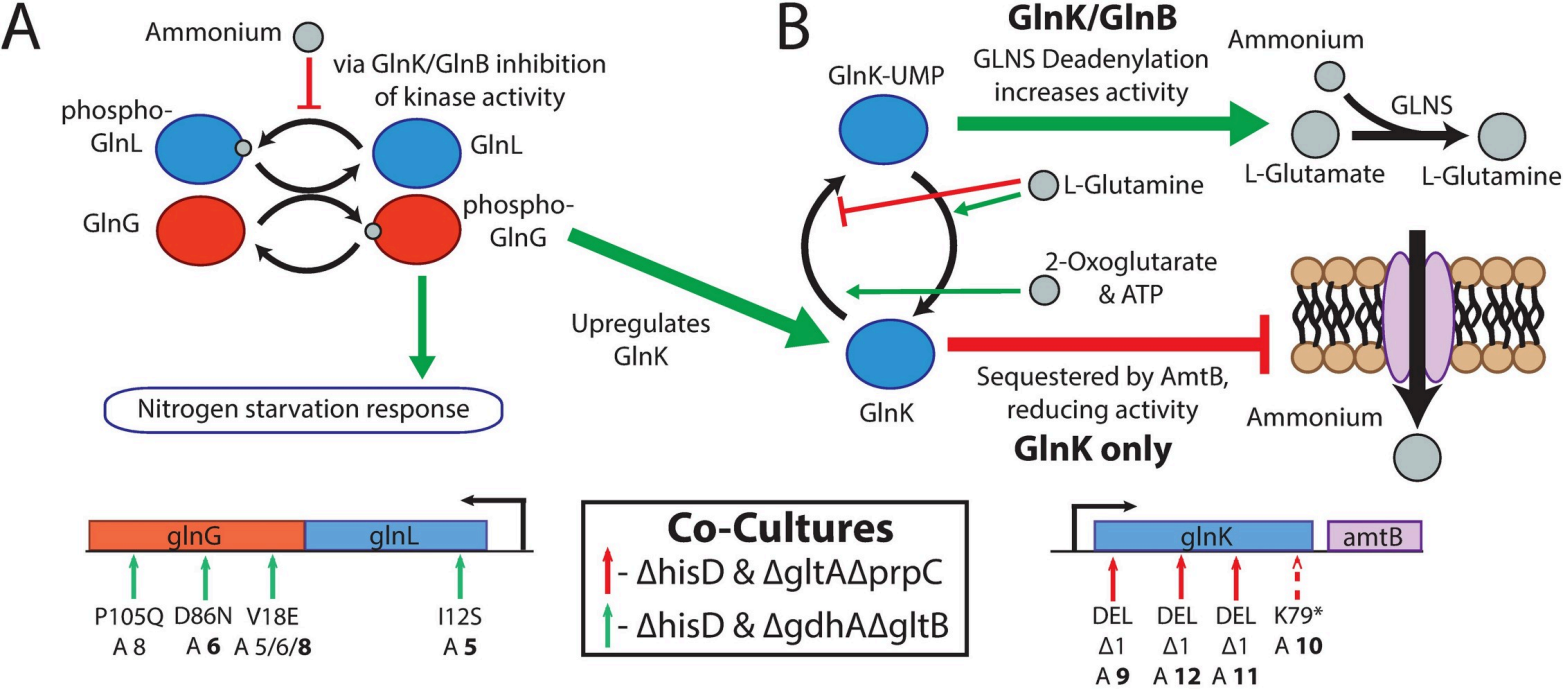
Mutations affecting nitrogen regulation. Functions of the mutated genes are summarized, and the location of all mutations are shown on the operon below the schematic. Mutations are shown if they appear at >10% frequency in more than one flask in an ALE lineage, and ALE numbers are in bold if the mutation appears in the endpoint clone. The mutations indicated with a dashed arrow occured in the Δ*hisD* strain and a solid arrow if they occured in Δ*hisD* strain’s partner MSE strain. (**A**) Mutations were acquired within the open reading frame of both genes comprising the nitrogen sensing two-component regulatory system. Shown in the schematic is the regulatory cascade in which nitrogen concentration is sensed (via GlnK or GlnB) by GlnL. In response to low nitrogen availability GlnL is autophosphorylated resulting in a subsequent transfer of the phosphorus group to GlnG. Phosphorylated GlnG upregulates general functions associated with nitrogen starvation, including increasing GlnK expression [77]. (**B**) Further, mutations were observed in the ORF of GlnK, one of two nitrogen metabolism regulators, sharing most functions with GlnB. Both genes become uridylylated in response to high concentrations of 2-oxoglutarate and ATP and low concentrations of L-glutamine, which is an indication of nitrogen limitation. GlnK-UMP can activate GLNS deadenylation, thus increasing its activity. Unlike GlnB, when GlnK is in a deuridylylated state (indicative of high nitrogen availability) it can be sequestered by the AmtB ammonium transporter reducing the transporter’s activity [75].

Knocking out enzymatic reactions in major biosynthetic pathways likely disrupts the homeostatic concentrations of key sensor metabolites, thus activating non beneficial stress responses (e.g., nutrient limited stress responses). The sequencing data was used to elucidate some of the adaptive mechanisms employed by the co-cultures following these pathway disruptions. For example, three frameshift deletions and a SNP resulting in a premature stop codon were observed early in the *glnK* ORF. These mutations were present in three Δ*gltA*Δ*prpC* endpoint clones and one Δ*hisD* endpoint clone from the Δ*hisD* & Δ*gltA*Δ*prpC* co-cultures (**Fig 6B**). GlnK along with GlnB are two nitrogen metabolism regulators with many overlapping functions. Both regulators are uridylated depending on the relative concentrations of 2-oxoglutarate, ATP, and L-glutamine. In conditions of high 2-oxoglutarate and ATP concentrations relative to L-glutamine concentrations, GlnK and GlnB are uridylated causing an increase in glutamine synthetase activity [75]. However, unlike GlnB, when GlnK is not uridylated it binds to the AmtB nitrogen uptake complex, thus reducing AmtB’s activity [76]. GlnK is also upregulated by GlnG of the nitrogen two-component regulatory system in the absence of nitrogen, unlike GlnB [77]. The citrate synthase knockout strain (Δ*gltA*Δ*prpC*) in particular could see a disruption in the homeostatic concentrations of metabolites immediately downstream of the citrate synthase reaction, including 2-oxoglutarate and L-glutamine. This could impair the ability of the cell to respond to sensors of nitrogen excess or limitation and respond with the appropriate global regulatory changes. Removing the activity of this GlnK mediated response system would prevent any detrimental cellular responses (such as inhibition of the AmtB nitrogen uptake complex) due to atypical concentrations of the sensor metabolites within the co-culture strains. No mutations were observed in the alternative nitrogen regulator, GlnB, throughout any of the evolutions.

Mutations found in the Δ*gdhA*Δ*gltB* strains imply a change in the activity of the two-component nitrogen regulatory system. The Δ*gdhA*Δ*gltB* strain in all Δ*hisD* & Δ*gdhA*Δ*gltB* lineages acquired mutations in the open reading frame of at least one gene in the two-component nitrogen regulator system, consisting of *glnG* (*ntrC*) and *glnL* (*ntrB*) (**Fig 6A**) [75]. Amino acid substitutions were observed in position 18, 86, and 105 of *glnG* corresponding to the response receiver domain of GlnG (based on protein families [78]), possibly augmenting GlnG’s ability to interact with GlnL. The endpoint clone of ALE #5 acquired an amino acid substitution of L-isoleucine to L-serine within a PAS domain of GlnL at position 12. This corresponds to the protein domain where regulatory ligands bind [79] suggesting this mutation could act to augment its activity in response to nitrogen availability. Like the citrate synthase knockout, the Δ*gdhA*Δ*gltB* strain would likely experience a change in the homeostatic concentrations of metabolites used to sense nitrogen availability. Thus, it can be hypothesized that the mutations observed in the nitrogen two-component regulatory system act to augment the expression of nitrogen uptake and assimilation processes regulated by GlnGL.

Mutations were also observed targeting osmotic stress responses and nonspecific stress responses. These are summarized in the **S1 Appendix**.

#### Genome duplications complement sequence changes

**Fig 7:**
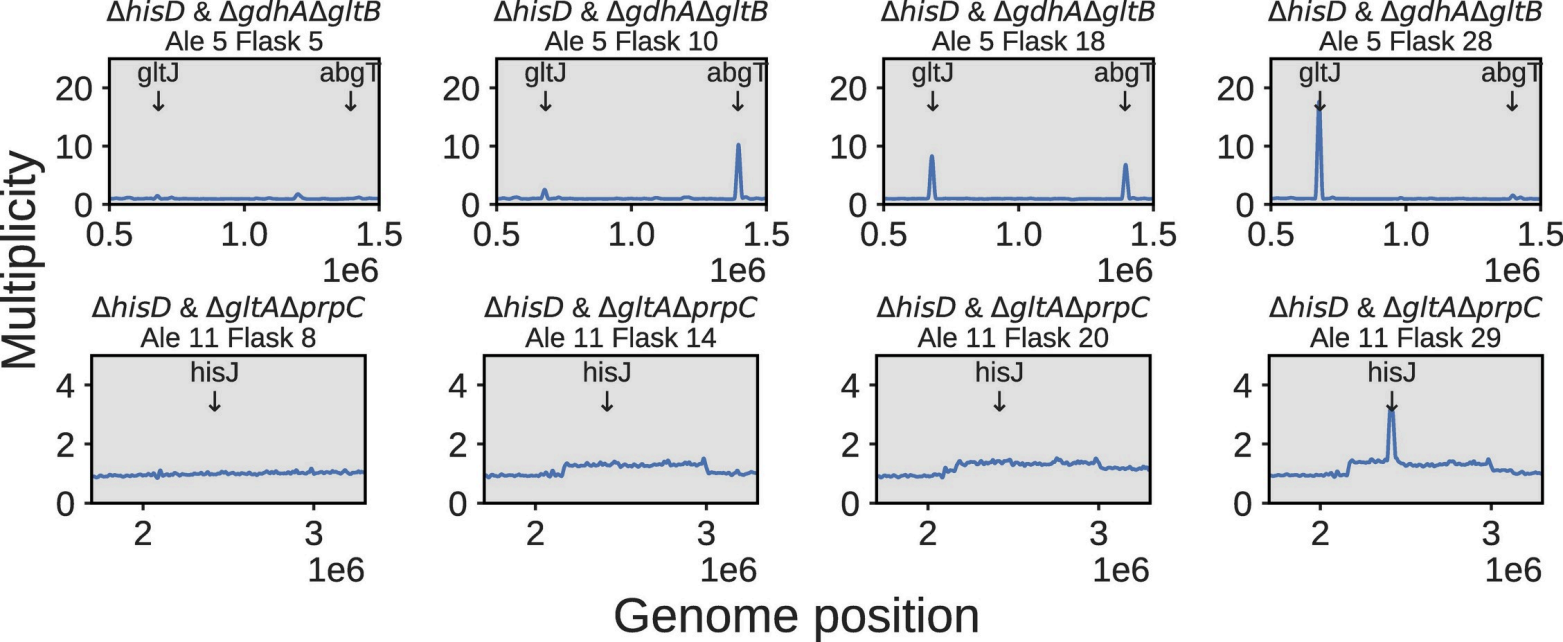
Duplication dynamics. The top panel depicts the dynamics of high multiplicity duplications in two transport complexes throughout the course of ALE #5 of a Δ*hisD* & Δ*gdhA*Δ*gltB* co-culture. A small region containing the *abgT* symporter of p-aminobenzoyl glutamate was duplicated early in the evolution, but later duplications in a region containing *gltJ*, along with the rest of the genes comprising the GltIKJL L-glutamate/L-aspartate ABC uptake system, became more prevalent. The bottom panel depicts the course of ALE #11, a Δ*hisD* & Δ*gltA*Δ*prpC* co-culture that initially showed a broad ~1 Mbp duplication. By the end of the evolution either a nested duplication emerged in a small genome region containing *hisJ*, along with the rest of the HisJMPQ L-histidine ABC uptake system, or a significant subpopulation emerged containing this duplication.

A complementary adaptive strategy for improving co-culture community growth was to acquire duplications in particular regions of the genome (**Figures H-J in S1 Appendix**). This evolutionary strategy possibly functioned in some cases to amplify expression of specific transporters to more efficiently uptake a metabolite that can rescue the strain’s auxotrophy (also observed in [80]). Alternatively, these duplications could function to provide genetic redundancy that increases the likelihood of acquiring mutations in the duplicated region [81,82]. For example, one of the three Δ*hisD* & Δ*gdhA*Δ*gltB* lineages displayed clear increases in sequencing depth near positions 674–683 kbp and 1,391–1,402 kbp, with multiplicities exceeding 15. The former of these coverage peaks contains 9 genes, including the 4 genes composing the GltIJKL L-glutamate/L-aspartate ABC uptake system [83]. The latter peak consisted of 10 genes including the 4 genes in the *abgRABT* operon, which facilitates the uptake of p-aminobenzoyl-glutamate and its hydrolysis into glutamate and 4-aminobenzoate [84]. This suggests that either L-glutamate, L-aspartate, or p-aminobenzoyl-glutamate could be cross-fed to the Δ*gdhA*Δ*gltB* strain *in vivo*. The *abgRABT* duplication, however, was depleted in favor of the *gltIJKL* duplication over the course of the evolution, suggesting L-glutamate or L-aspartate is the preferred cross-feeding metabolite over p-aminobenzoyl-glutamate (**Fig 7, Table 2**).

While the duplications mentioned above presented clear amplifications in targeted operons, some observed duplications consisted of 100,000s of basepairs and 100s of genes. Further, many of the duplications seen in the populations were not observed in the resequenced endpoint clones. Possible explanations for these observations can be found in the **S1 Appendix**.

### Modeling community features of auxotroph communities

Community genome-scale models were applied to understand the basic characteristics of the co-culture communities generated in this study. Given the growing appreciation for the role of limited protein availability on governing many fundamental *E*. *coli* growth characteristics [36], community genome-scale models of metabolism and gene expression (ME-models) were utilized. A new computational approach was also developed, as a community modeling method did not exist that was suitable for studying co-cultures growing in an ALE experiment while also being amenable to ME-models (see **Methods**).

**Fig 8:**
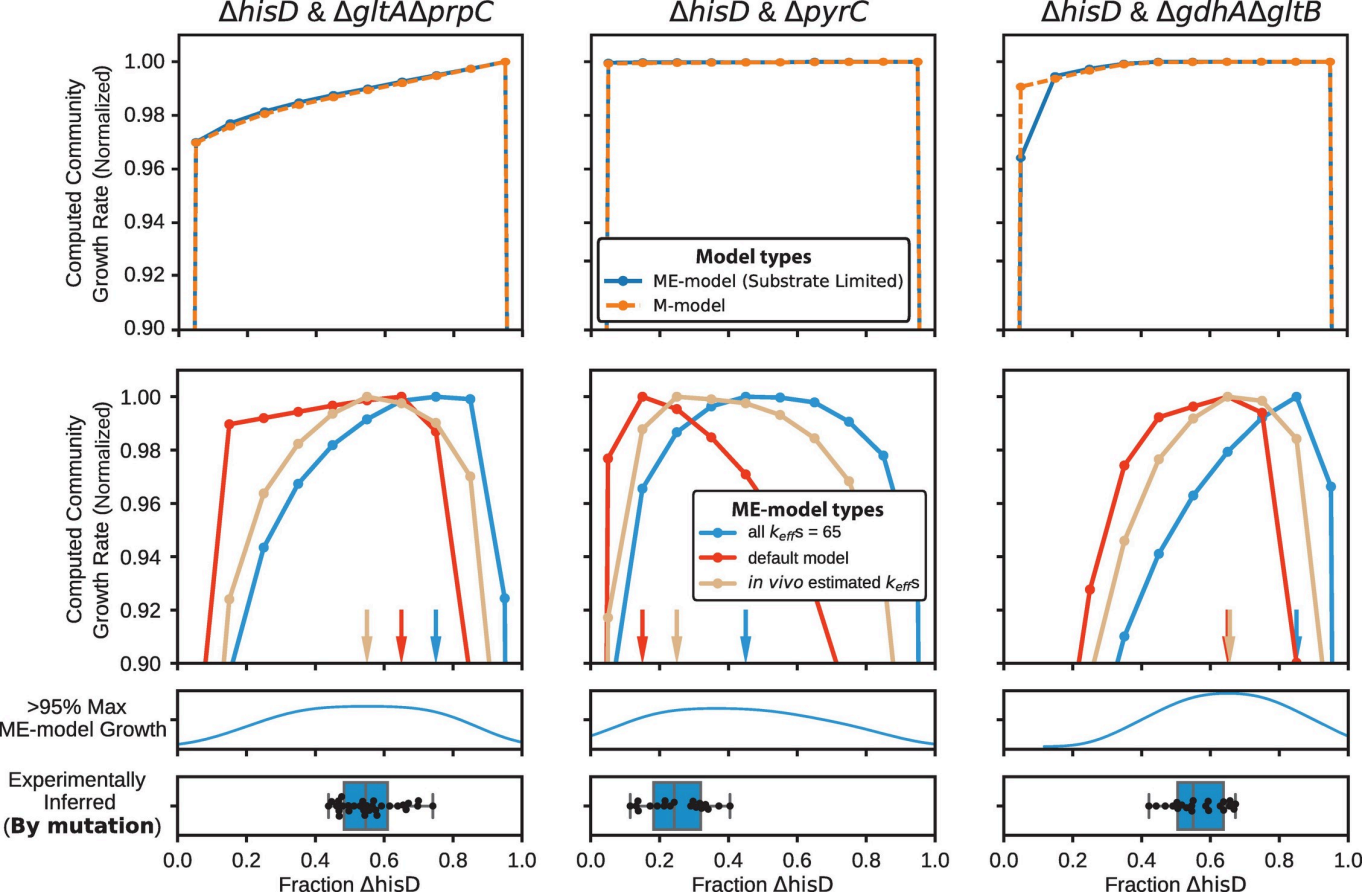
Comparison of community M- and ME-models. The simulated growth rates for fractional strain abundances of Δ*hisD* ranging from 0 to 1. The top panel shows the community growth rate predictions of the community M-model and the community ME-model simulated in glucose-limited *in silico* conditions. The bottom panel shows growth rate predictions for the community ME-model simulations in glucose excess conditions. The arrows correspond to the fractional abundance that provided the highest computed community growth rate. The fractional abundances with growth rates greater than 95% of the maximum computed value were represented as a kernel density plot. The high density regions of the kernel density plot aligned well with the experimentally inferred community compositions, shown in the box plot.

Using community M- and ME-models, the role of substrate and proteome limitations on basic community characteristics was assessed. To that end, both types of community models were constrained to uptake no more than 5 mmol gDW_community_^-1^ hr ^-1^ of glucose and simulated over a fractional Δ*hisD* strain abundance of 0 to 1 (**Fig 8)**. The communities were allowed to cross-feed any metabolite that could restore growth in the partner strain (**Table D in S1 Appendix**). At this low glucose uptake rate the community ME-model was being simulated in the so-called substrate-limited region [37], meaning that the community growth rate was determined solely by the amount of substrate available. In this region the protein allocation constraints inherent in the ME-model were mostly inactive. In the substrate-limited region, the ME-model and M-model behaved similarly and predicted little change in the community growth rate regardless of the fractional abundance of the strains in co-culture. Alternatively, the community ME-model was again simulated, but with an unlimited amount of glucose available to the *in silico* community. These simulations therefore occurred in the proteome-limited region of the community ME-model, meaning that the growth rate was determined by limitations in the protein available to carry out their enzymatic functions. When simulating the community ME-model in the proteome-limited region, notable composition-dependent variation in the community growth rate was observed across all fractional strain abundances (**Fig 8**). Metabolite exchange for substrate- and proteome-limited ME-models was also observed (**Figures M-N in S1 Appendix**)

ME-model predictions are dependent on parameters that couple protein abundance to the flux values of the processes or reactions that they catalyze. These are called “k_eff_s” and are analogous to the effective *in vivo* turnover rate of an enzyme. Obtaining these values on a genome-scale is a notoriously difficult problem [85], and no “gold standard” set of k_eff_s currently exists. To account for uncertainty in these k_eff_ parameters, proteome limited community ME-model simulations were repeated using three different k_eff_ sets, including one set of naive values (“all k_eff_s = 65”) and two sets derived using experimental data (“default model” [86] and “*in vivo* estimated k_eff_s” [87,88]). All fractional abundance values within 95% of the maximum community growth rate were compiled and represented as a kernel density plot. The computed optimal community compositions (i.e., strain ratios that enabled the fastest computed community growth) showed relatively good agreement with the experimentally inferred community compositions (**Fig 8**). See the **Methods** for a description of the three k_eff_ sets.

**Fig 9:**
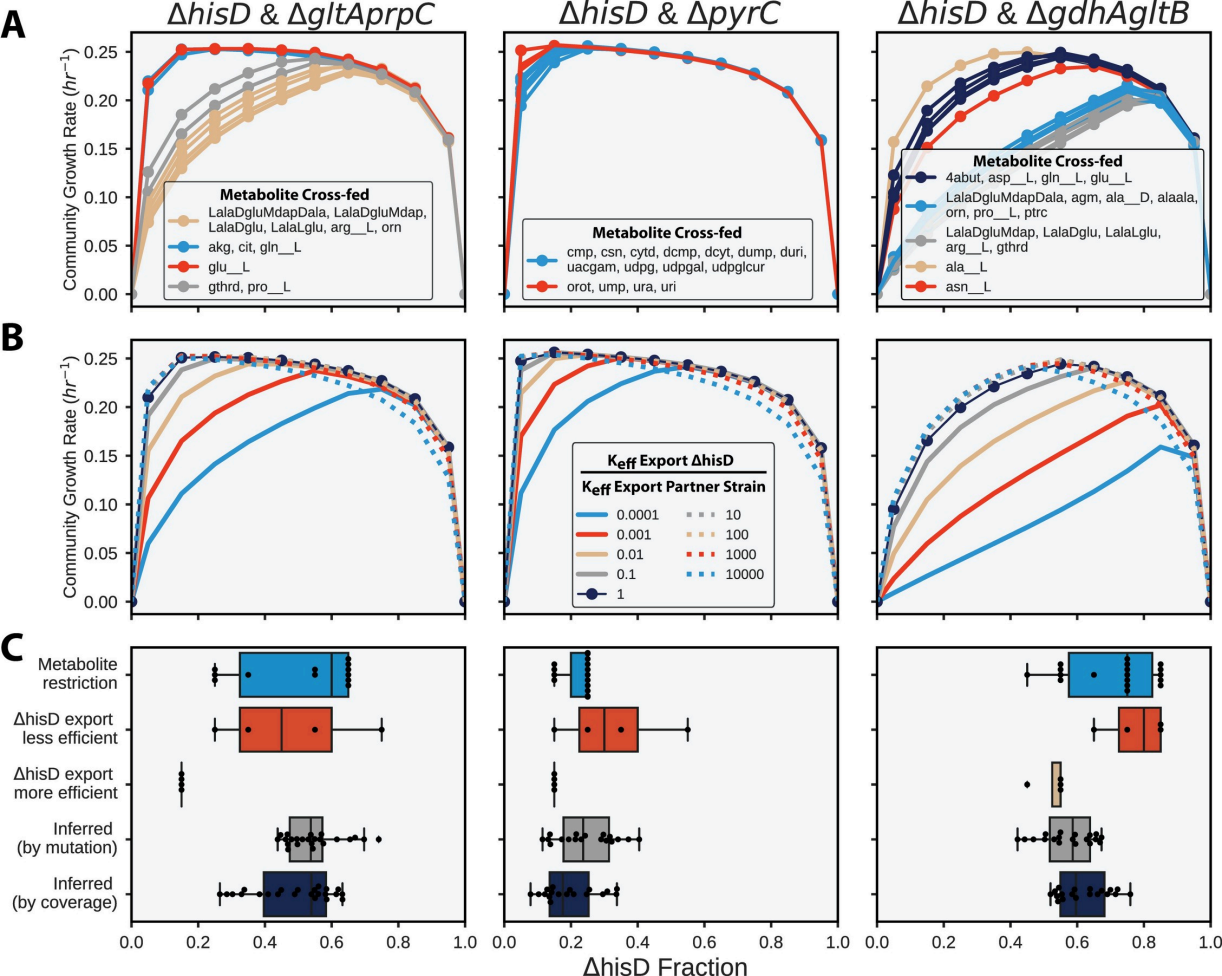
Community modeling. Community ME-model-predicted growth rates computed with fractional strain abundances of Δ*hisD* ranging from 0 to 1. (**A**) The effect of metabolite cross-feeding on community structure. Each curve was computed after allowing each of the metabolites in the legend to exclusively be cross-fed to the MSE strain. Curves with identical computationally-predicted optimal strain abundances were grouped and given the same color. (**B**) The effect of varying the proteome efficiency of metabolite export on community structure (see **Methods**). The analysis was performed on models constrained to only cross-feed the metabolite that was considered most likely to be cross-fed to the Δ*gltA*Δ*prpC*, Δ*pyrC*, and Δ*gdhA*Δ*gltB* strains *in vivo* based on the sequencing data (2-oxoglutarate, orotate, and L-glutamate, respectively) (**Table 2**). (**C**) Box plots of experimentally inferred fractional strain abundances for each sample (bottom two rows, gray and dark blue) and the computationally-predicted optimal strain abundances following variation in the cross-feeding metabolite (top row, blue) and in strain proteome efficiency (second and third row, red and yellow).

The ME-modeling analysis suggested that it may be necessary to consider protein allocation when studying co-culture evolutions, therefore necessitating the use of resource allocation models, such as ME-models. The community ME-models thus were used to predict how the community composition could vary depending on basic characteristics of the co-cultures: 1) the identity of the metabolite that is cross-fed or 2) the enzyme efficiency of the community members. These simulations predicted that the metabolite being cross-fed within the community could have a sizeable impact on both the community composition and growth rate. This is particularly true for the Δ*hisD* & Δ*gdhA*Δ*gltB* and Δ*hisD* & Δ*gltA*Δ*prpC* simulations which showed that metabolite cross-feeding affected the growth rate and community compositions by as much as 50% (**Fig 9A**).

The strains growing in co-culture *in vivo* each undoubtedly differed in the protein cost required to synthesize the metabolite required by its partner strain. Therefore a proteome efficiency analysis (see **Methods**) was performed which showed that the computed optimal community compositions (the fractional strain abundance that gave the maximum community growth rate) of all three co-cultures were sensitive to the strain’s efficiency (**Fig 9B**). The computed optimal community composition was most sensitive when the Δ*hisD* strain’s metabolite export was less proteome efficient than its partner MSE strain. This observation is not surprising given that the Δ*hisD* strain must secrete metabolite(s) to the MSE strain at a much higher flux than the MSE strain to the Δ*hisD* strain. Therefore, a decrease in protein efficiency will have a larger impact on the Δ*hisD* strain. The community models also unintuitively predicted that, if the Δ*hisD* strain required a greater protein investment to produce the metabolite required by the partner strain (i.e., if the Δ*hisD* strain was less efficient than its partner), the abundances of the Δ*hisD* strain would actually increase in the community.

The optimal predicted community composition for the two above computational analyses shown in **Fig 9A and 9B** are summarized in **Fig 9C**. The figure shows general agreement between the computed optimal community compositions and the experimentally inferred community composition, even after varying key features of the community simulation (metabolite cross-feeding and protein efficiency). This suggests that community ME-models have the potential to be useful tools for understanding the behavior of simple communities. The same analysis was performed with the “*in vivo* estimated k_eff_s” set of k_eff_s and showed similar behavior (**Figure O in S1 Appendix**).

## Discussion

This work provides genetic-level insight into the adaptation of model-designed nascent syntrophic communities growing cooperatively in suspension. This effort produced a novel algorithm, called OptAux, which was validated against historical auxotrophs and used to predict novel auxotrophic strain designs. OptAux-predicted designs with diverse metabolic deficiencies were co-cultured and community growth was optimized via adaptive laboratory evolution. Sequencing these co-cultures throughout the evolutions gave mutation and community composition information, thus providing insight into mechanisms of cellular cooperation. An additional modeling method was developed to interpret community features and demonstrated the importance of considering protein synthesis cost when studying cooperative communities in the utilized experimental conditions.

OptAux was demonstrated to be a useful tool for designing new types of cellular auxotrophies. Unlike many previously studied auxotrophies, OptAux enabled the prediction of auxotrophs stemming from a diverse set of major metabolic deficiencies. This included the prediction of 4 potential new essential biomass component elimination (EBC) designs and 20 unique major subsystem eliminations (MSE) designs. The OptAux-predicted MSE strains themselves could reveal further community insights if studied in co-culture. Co-cultures of two MSE strains would likely require a significant degree of metabolic rewiring in each strain to form a viable microbial community, thus probing the alternate evolutionary and cooperative paths such complex combinations could produce. OptAux is also suitable for predicting new auxotrophies in any organism outside of *E*. *coli*, provided the organism has an existing metabolic reconstruction [89].

Sequencing co-cultures throughout the course of the evolution experiments offered insight into the major adaptive mechanisms underlying the evolution of microbial cooperativity. The observed mutations indicated two major adaptive strategies employed by the strains in co-culture 1) mutating transporters, likely to improve uptake of auxotrophic metabolites (**Fig 5**) and 2) mutating to adapt to homeostatic changes as a result of metabolic disruptions upon imposing gene knockouts (**Fig 6**). The reported transporter mutations could prove useful for metabolic engineering applications, as optimizing the metabolite uptake characteristics of transporters can be an important component of improving the performance of engineered strains [90]. There, however, were no observed mutations, outside of mutations in a predicted GABA:L-glutamate antiporter in a Δ*hisD* strain, hinting at how the strains were capable of rewiring their intracellular metabolism to supply their partner strain with the required metabolite (i.e., no observed mutations associated with biosynthetic pathways). A future direction of this work could be to further evolve these strains to observe if new mutations appear to enhance metabolite rewiring. Alternatively, it is possible that the co-cultures grew by clumping and employing nanotube-mediated cross-feeding [91], which may be explored using microscopy.

Community ME-models were applied to understand the factors that drive community composition. This was the first community modeling effort to demonstrate the necessity of considering protein allocation when computationally studying community features. Interestingly, some of the studied co-cultures evolved to consistent community compositions that skewed away from a 50:50 strain ratio, a feature the community ME-models were often capable of capturing (**Fig 8**). Additionally, the community ME-models predicted that, if the Δ*hisD* strain became less protein efficient at producing the necessary cross-feeding metabolite, the optimal abundance of the Δ*hisD* strain in the co-culture would actually increase (**Fig 9**). Though unintuitive, this prediction is in agreement with a paradox highlighted in a previous computational study of community dynamics [92].

Despite the observed agreement between measured and computed optimal community compositions, this work highlighted the fact that there are a vast number of variables that could potentially influence basic features of simple communities. Experimentally assessing important features such as metabolite cross-feeding and community structure—as touched on here—on a large scale with many different cohorts and combinations is necessary to adequately understand the behavior of such bacterial communities. Model-driven design of communities and the use of community ME-models, however, present a more complete computational framework that can be leveraged as a tool to extract more knowledge from such experiments. Further, community ME-models offer a means to probe how factors outside of metabolism (e.g., translation efficiency and proteostasis) could affect community characteristics.

## Materials and methods

### Computational methods

All constraint-based modeling analyses were performed in Python using the COBRApy software package [93] and the *i*JO1366 metabolic model of *E*. *coli* K-12 MG1655 [49]. All optimizations were performed using the Gurobi (Gurobi Optimization, Inc., Houston, TX) mixed-integer linear programming (MILP) or linear programming (LP) solver. The community ME-models were solved using the qMINOS solver in quad precision [94,95]. All scripts and data used to create the presented results can be found at www.github.com/coltonlloyd/optaux.

#### OptAux algorithm formulation

For the presented work it was necessary to employ an algorithm capable of finding reaction knockouts that would ensure the target metabolite is computationally essential in the *in silico* growth media for all feasible growth rates. To this end, a new algorithm was written as opposed to implementing a “reverse” version of RobustKnock (i.e., RobustKnock where the target objective is metabolite uptake instead of secretion). A “reverse” RobustKnock implementation would optimize the minimum required uptake of a metabolite *at* the maximum growth rate, thus leading to strain designs that must uptake a high amount of the target metabolite only when approaching the maximum growth rate (**Figure A in S1 Appendix**). To prevent this computational phenotype with OptAux, the inner problem optimizing for growth rate, which was utilized in RobustKnock, was removed. The growth rate was instead constrained to the *set_biomass* value, thus forcing the optimization to occur at a predefined growth rate. The constraint was implemented by setting the upper and lower bounds of the biomass objective function to *set_biomass*. Using relatively low *set_biomass* values with OptAux ensured the target metabolite would be computationally required for all feasible growth rates. For the simulations ran in this study (**S1 Data**), the *set_biomass* value was set to 0.1 hr ^-1^.

An additional constraint was included in OptAux to represent additional metabolites present in the *in silico* media that could alternatively be used for growth, called the *competing_metabolite_uptake_threshold*. It was applied by finding all metabolites with exchange reactions and a default lower bound of 0 mmol gDW ^-1^ hr ^-1^ and increasing the bound to the *competing_metabolite_uptake_threshold*, thus allowing alternative metabolites in the *in silico* media to compete for uptake with the target metabolite. Increasing this threshold ultimately increases the specificity of the OptAux solution (i.e., whether other metabolites could potentially restore growth in addition to the target metabolite). In other words, if other metabolites were present in the *in silico* media, would the model still be auxotrophic for the target metabolite? If the strain would still be auxotrophic, it can be said to have high specificity; if the strain would not be auxotrophic, it can be said to be non-specific or semi-specific.

The resulting OptAux algorithm is a bilevel MILP (**Fig 2B**) that can be found at www.github.com/coltonlloyd/optaux.

#### OptAux simulations

The OptAux algorithm was ran for all carbon containing metabolites with exchange reactions in *i*JO1366. The model’s default glucose M9 minimal *in silico* media was used for all optimizations with the maximum oxygen uptake set to 20 mmol gDW ^-1^ hr ^-1^. For each optimization the target metabolite was selected, and the maximum uptake of the metabolite was set to 10 mmol gDW ^-1^ hr ^-1^. The model was then reduced by performing flux variability analysis (FVA) on every reaction in the model and setting the upper and lower bounds of each reaction to the FVA results. If FVA computed that no flux could be carried through the reaction, then it was removed from the model. Additionally, reactions were excluded from knockout consideration if they met one of the following criteria: **1)** it was an *i*JO1366 false positive when glucose is the primary carbon substrate [96] **2)** it was essential in LB rich media [15] **3)** its annotated subsystem was one of the following: Cell Envelope Biosynthesis, Exchange, Inorganic Ion Transport and Metabolism, Lipopolysaccharide Biosynthesis / Recycling, Murein Biosynthesis, Murein Recycling, Transport, Inner Membrane, Transport, Outer Membrane, Transport, Outer Membrane Porin, or tRNA Charging **4)** it involved a metabolite with more than 10 carbons **5)** it was a spontaneous reaction.

#### Identifying gene mutations and duplications

The FASTQ data from the sequencing samples was filtered and trimmed using AfterQC version 0.9.6 [97]. The quality controlled reads were aligned to the genome sequence of *E*. *coli* K-12 BW25113 (CP009273.1) [98] using Bowtie2 version 2.3.0 [99]. Mutations were identified based on the aligned reads using breseq version 0.32.0b [65]. If the sample was of a co-culture population and not a clone, the predict polymorphism option was used with a frequency cutoff of 0.025. The output of the breseq mutation analysis for all samples can be found in **S3 Data** and on www.aledb.org [100].

Duplications were found by analyzing the BAM sequence alignment files output from Bowtie using the pysam Python package [101]. Pysam was used to compute the sequencing read depth at each DNA position within the genome sequence. For population samples, a cutoff of 1.25 x coverage fit mean (a measure of average read alignment coverage over the genome) was used. This relatively low threshold was used to account for the varying fractional abundances of the strains in community. A gene was flagged as duplicated in the sample if over 80% of the base pairs in the gene’s ORF had alignment coverage above the duplication threshold. Duplications found in starting strains were excluded from the duplication analysis. Further, the set of duplicated genes were grouped together if they were located next to each other on the genome. A new group was created if there existed more than five genes separating a duplicated gene from the next duplicated gene in the genome (**S4 Data**).

Aligned read coverage across the *E*. *coli* genome is noisy and therefore was filtered before plotting in order to observe its dominant features. This was accomplished by first splitting the coverage vector into 50,000 segments, such that each segment represented ~100 base pairs, and the average of the segments was found. Locally weighted scatterplot smoothing (LOWESS) was then applied to the array of concatenated segments using the statsmodel package in python [102]. For the smoothing, 0.5% of all of the segments was used when estimating each coverage value (y-value), and zero residual-based reweightings were performed. The remaining parameters were set to their default.

#### Calculating strain abundances from sequencing data

The fractional abundances of the strains in co-culture were predicted using two features of the sequencing data obtained from each co-culture sample: **1)** the frequency of characteristic mutations of each strain and **2)** the read depth of the knocked out genes.

Each of the stains used in this study possessed a unique characteristic mutation (**Table C in S1 Appendix**), which could be used as a barcode to track the strain. The breseq mutation calling pipeline identified the characteristic mutations of each strain in co-culture and reported the frequency that the mutation was observed. This information was thus used to track the strain’s presence. For strains with two characteristic mutations (e.g., Δ*hisD* and Δ*gdhA*Δ*gltB*) the reported frequency of the genes was averaged and used as a prediction of the relative abundance of that strain. One mutation in particular, an IS element insertion in *yqiC*, which is characteristic of the Δ*hisD* strain, was not detected in several samples when Δ*hisD* was in co-culture with Δ*pyrC*. This is likely due to the low abundance of the Δ*hisD* strain in that particular population. In those cases, the Δ*hisD* strain’s abundance was predicted using only the frequency of the *lrhA*/*alaA* intergenic SNP (**Figure F in S1 Appendix**). For one sample (A10 F23 I1 R1) the sequencing coverage was too low (~14.5) and the Δ*gltA*Δ*prpC* characteristic mutation was not detected. Therefore no relative abundance was computed for this sample.

The second method for computing fractional strain abundances used the sequencing read alignment to compare the coverage of the deleted genes in each strain to the average coverage of the sample. As an example, for a strain paired with the Δ*hisD* strain, the average coverage of the base pairs in the *hisD* ORF divided by the average coverage for that sample, would give an approximation of its relative abundance in the population. As with the characteristic mutation approach, if the two genes were knocked out in the strain, the average coverage of the two genes was used to make the approximation (**Figure E in S1 Appendix**).

When reporting the relative abundance predictions (**Figs 8 and 9**), the computed abundances of each strain were normalized by the sum of the computed abundances of the two strains in co-culture. This ensured that the abundance predictions summed to one. Predictions made using the two described methods showed general agreement (**Figure F in S1 Appendix**).

#### Community modeling

A community modeling approach was formulated that was amenable to ME-models and consistent with the characteristics of the ALE experimental design. The ALE experimental design applies a constant growth rate selection pressure by ensuring the cells are maintained in exponential growth phase in nutrient excess media conditions. A consequence of this experimental design when applied to co-culture systems is that the strains in co-culture must be growing at the same growth rate, on average. If this was not the case, one strain would be diluted from the culture or there would be dramatic fluctuations in the community composition, which is not the case (**Fig 9C**). Further, ALE experiments ensure that the culture is well mixed and grown in an excess of nutrients. These experimental conditions are not amenable to most existing community modeling methods. One modeling framework exists to study communities growing in steady state, called SteadyCom [23] (**Figure L in S1 Appendix**), though this method is not compatible with ME-models. This is due to the ME-model’s use of non-linear macromolecular coupling constraint expressions that are formulated as a function of growth rate. Therefore, the conversion to “aggregate biomass” flux used in the SteadyCom formulation cannot be translated directly to ME-models.

Given the above considerations, a multicompartment FBA approach, similar to community FBA [26] was used where the growth rates of the co-culture strains were constrained to be equal. The community model included one compartment for each of the two mutant strains in co-culture and a shared compartment where each of the strains could exchange metabolites. Further, the fluxes in and out of each strain’s compartment were scaled by the strain’s relative abundance to effectively mass balance the different model compartments (**Figure K in S1 Appendix**), thus allowing the relative abundance of each strain to be imposed as a parameter. For secretion, this was done by multiplying these exchange reactions as follows:

$${metabolite}_{Strain\;1}\overset{V_{secrete}}{\to }X_{Strain\;1}\cdot {metabolite}_{Shared}$$

and for uptake:

$$X_{Strain\;2}\cdot {metabolite}_{Shared}\overset{V_{uptake}}{\to }{metabolite}_{Strain\;2}$$

where v_secrete_ is the secretion flux from strain 1 and has units of mmol gDW_Strain1_^-1^ hr ^-1^ and X_Strain1_ is the fractional abundance of strain 1 with units of $\frac{{gDW}_{Strain1}}{{gDW}_{Community}}$. Therefore, applying this coefficient to metabolite_Shared_ gives fluxes in the shared compartment units of mmol gDW_Community_^-1^ hr ^-1^. For the subsequent uptake of the shared metabolite by strain 2, the fractional abundance of strain 2 is applied giving units of mmol gDW_Strain2_^−1^ hr ^-1^ (**Figure K in S1 Appendix**).

Using this community modeling approach, the fractional abundance of each strain in the co-culture was implemented as a parameter that could be varied from 0 to 1, which in turn had an impact on the optimal growth state of the community. All presented simulations were ran by optimizing the community growth rate for 10 values of X_Strain1_ (abundance of strain 1) ranging from 0.05 to 0.95. For X_Strain1_ values of 0 or 1 the community growth rate was assumed to be 0 hr ^-1^ given that the co-culture mutants are auxotrophic and require the presence of both mutants to grow. The metabolites that were allowed to be cross-fed in simulation were limited to the set of metabolites that can computationally restore the growth of each auxotroph mutant (**Table D in S1 Appendix**).

For the community simulations, the *i*JL1678b [39] ME-model and *i*JO1366 [49] M-model of *E*. *coli* K-12 MG1655 were used. For proteome-limited ME-models simulations, the uptake of metabolites in the *in silico* glucose minimal growth media into the shared compartment was left unconstrained, as the ME-model is self-limiting [37]. For glucose-limited ME-model and M-model simulations, the maximum glucose uptake into the shared compartment was constrained to 5 mmol gDW_Community_^-1^ hr ^-1^. The non-growth associated ATP maintenance and the growth associated ATP maintenance were set to the default parameter values in the model. For ME-model simulations, the RNA degradation constraints were removed to prevent high ATP costs at the low community growth rates. Since the newly formed communities are unoptimized and growing slowly, the ME-model’s unmodeled/unused protein fraction parameter was set to a higher value, 0.75, for proteome limited simulations (an unmodeled/unused protein fraction of 0.65 was imposed when the “*in vivo* estimated k_eff_s” parameter set was used, since these k_eff_s give a lower maximum growth rate than the other two k_eff_ vectors used) and the default value, 0.36, for glucose-limited simulations. If a metabolite had a reaction to import the metabolite across the inner membrane but no export reaction, a reaction to transport the metabolite from the cytosol to the periplasm was added to the model. For more on the ME-model parameters, refer to [39] and [37].

Three different sets of enzyme turnover rates (k_eff_s) were used for the community ME-model simulations (**Fig 8**). The first set of k_eff_s (“all k_eff_s = 65”) was imposed by setting all k_eff_s in *i*JL1678b-ME equal to 65 s^-1^. The next set of k_eff_ values (“default model”) used the default set of k_eff_ parameters included with *i*JL1678b-ME. Most of the metabolic k_eff_s in this default set are determined by scaling a median k_eff_ value (65 s^-1^) by an estimation of the solvent accessible surface area of the enzyme complex that catalyzes the reaction (reference [37] for further description). The default k_eff_ parameters further included a set of 284 metabolic k_eff_s derived using proteomics data and a computational method developed in Ebrahim *et al*. [86]. The last k_eff_ set (“*in vivo* estimated k_eff_s”) included 234 k_eff_s from Davidi *et al*. [87] that were estimated using model-computed fluxes and proteomics data. The k_eff_s not estimated in Davidi *et al*. were imputed using the median estimated k_eff_ value from Davidi *et al*. (6.2 s^-1^). For all three k_eff_ sets, all non-metabolic processes were assigned a k_eff_ of 65 s^-1^.

Assessing the influence of metabolite cross-feeding on community composition was performed by restricting the simulation to cross-feed only one of the metabolites computationally predicted to restore growth in the MSE strain. In doing so, the identity of the metabolite being cross-fed could be related to the optimal community growth rate and structure.

To vary the proteome efficiency (k_eff_) of secreting the cross-fed metabolites, first the exchange reactions into the shared compartment for all potential cross-feeding metabolites were constrained to zero, except the metabolite inferred from the experimental data (**Table 2**). Then the enzymatic efficiency of the outer membrane transport process of the inferred cross-feeding metabolite was altered in each strain. The outer membrane transport reactions for each inferred metabolite (i.e., HIStex, GLUtex, AKGtex, and OROTtex for L-histidine, L-glutamate, 2-oxoglutarate, and orotate, respectively) have multiple outer membrane porins capable of facilitating the transport process. To account for this, the k_eff_ kinetic parameter of each porin and reaction was changed by multiplying the default k_eff_ value by the appropriate multiplier. The COBRAme software was used for all ME-model computations [39].

#### Reproducibility

All code and data necessary to reproduce the presented results can be found on GitHub at https://github.com/coltonlloyd/OptAux.

### Experimental methods

#### *E*. *coli* strain construction

All single gene knockouts used in this work were obtained from the Keio collection, a collection of all single gene knockouts in *E*. *coli* K-12 BW25113 [15]. To generate double gene knockout strains, the second knockout genes were identified from the Keio collection as donor strains, and their P1 phage lysates were generated for the transduction into the receiving single knockout strains. For instance, the Δ*gltA* or Δ*gltB* knockout strain was a donor strain and the Δ*prpC* or Δ*gdhA* knockout strain was a receiving strain (**Table B in S1 Appendix**), respectively. These four knockout strains were used for the construction of the double knockout strains, Δ*gltA*Δ*prpC* and Δ*gdhA*Δ*gltB*. Each mutant was confirmed not to grow in glucose M9 minimal media without supplementation of an auxotrophic metabolite predicted by the *i*JO1366 model.

#### Adaptive laboratory evolution

Knockout mutants were each initially grown in lysogeny broth from a single colony, then washed 3 times and resuspended in M9-4g/L glucose medium. The washed cells from each knockout mutant preculture were then transferred to fresh M9-4g/L glucose medium and co-cultured with mutants from the partner strain. Cultures were initially inoculated with equal numbers of cells from the two relevant auxotrophs, then serially propagated (100 μL passage volume) in 15 mL (working volume) flasks of M9 minimal medium with 4 g/L glucose, kept at 37°C and well-mixed for full aeration. An automated system passed the cultures to fresh flasks once they had reached an OD600 of 0.3 (Tecan Sunrise plate reader, equivalent to an OD600 of ~1 on a traditional spectrophotometer with a 1 cm path length), a point at which nutrients were still in excess and exponential growth had not started to taper off. Four OD600 measurements were taken from each flask, and the slope of ln(OD600) vs. time determined the culture growth rates. The timescale of the evolution was reported using the cumulative number of cell divisions, as opposed to generations or days, as mutations occur primarily during cell division events [64].

#### Resequencing

Co-culture population samples were collected at multiple midpoints throughout the ALE and sequenced. Additionally, the starting mutant strains and clones of both mutants isolated from the ALE endpoints were sequenced. The Δ*hisD* endpoint clone was unable to be isolated via colony selection for ALE #11. Genomic DNA of the co-culture populations and mutant clones was isolated using the Macherey-Nagel NucleoSpin tissue kit, following the manufacturer’s protocol for use with bacterial cells. The quality of isolated genomic DNA was assessed using Nanodrop UV absorbance ratios. DNA was quantified using the Qubit double-stranded DNA (dsDNA) high-sensitivity assay. Paired-end whole genome shotgun sequencing libraries were generated using KAPA HyperPlus kits and run on an Illumina MiSeq platform with a PE600v3 kit or an Illumina HiSeq 4000 with a PE-410-1001 kit for 150bp reads. DNA sequencing data from this study is available on the Sequence Read Archive database (accession no. SRP161177).

## Supporting information

S1 Data OptAux solutions. Output of the OptAux algorithm ran for one, two, and three reaction knockouts on glucose minimal media for all carbon containing exchange metabolites. Four different competing metabolite uptake thresholds were used (0, 0.01, 0.1, 2).
(XLSX)

S2 Data Major subsystem elimination designs. All MSE designs along with further information regarding the subsystems of the reaction knockouts and the metabolites that can restore growth in each design.
(XLSX)

S3 Data Mutations. The breseq identified mutations for all samples collected in this work. Both the full output and a table with only mutations observed in the endpoint clones are provided.
(XLSX)

S4 Data Duplications. Genes with read coverage meeting the duplication criteria. Separate spreadsheets are provided for all samples using the mutant pair, ale number, flask number, isolate number, and replicate number to identify each sample.
(ZIP)

S1 Appendix Supplemental text and figures. (PDF)
