# Supplementary material for: The genetic basis for adaptation of model-designed syntrophic co-cultures
Source: PLoS Comput Biol. 2019 Mar 1;15(3):e1006213. doi: 10.1371/journal.pcbi.1006213 (PMC6415869; doi:10.1371/journal.pcbi.1006213)
Supplement: S1 Appendix — (PDF) [file pcbi.1006213.s005.pdf]

# Supplemental Text and Figures

**Supplement Figures**

1. Figure A: OptAux Categories toy example
2. Figure B: Network cartoons for MSE and EBC
3. Figure C. Metabolite uptake demand for the two OptAux solution types.
4. Figure D: ALE trajectories
5. Figure E: Relative abundance predictions coverage/characteristic
6. Figure F: Comparison of abundance predictions using both methods
7. Figure G: Mutations observed associated with the general *E. coli* stress response
8. Figure H: Duplications ΔhisD & ΔgltAΔprpC
9. Figure I: Duplications ΔhisD & ΔgdhAΔgltB
10. Figure J: Duplications ΔhisD & ΔpyrC
11. Figure K: Community ME-modeling overview
12. Figure L: Comparison between community M- model and SteadyCom predictions
13. Figure M: ME-model simulation metabolite exchange for all fractional abundances
14. Figure N: Optimal cross-feeding of ME-model simulation when limited to experimentally inferred metabolite.
15. Figure O: ME-model simulation metabolite exchange for only experimentally inferred metabolite

**Supplement Tables**

1. Table A: EBC Designs and validation, if available
2. Table B: Mutant Strain Summaries
3. Table C: Characteristic Mutations
4. Table D: Restorative metabolite for each strain evolved in co-culture
5. Table E: ΔhisD & ΔgltAΔprpC endpoint clone mutations
6. Table F: ΔhisD & ΔgdhAΔgltB endpoint clone mutations
7. Table G: ΔhisD & ΔpyrC endpoint clone mutations

# Supplemental Text

## Description of OptAux Solutions

**Essential Biomass Component Elimination**

Essential biomass component elimination (EBC) designs generated using OptAux highlight traditional approaches to designing an auxotroph strain. To do this, EBC designs typically targeted reactions directly upstream of the synthesis of biomass components. As shown in **Fig 3A**, all pathways that can be used by the network to produce L-asparagine are knocked out. As a consequence, this strain requires supplemental L-asparagine, with no alternative, in order to sustain growth, thus constituting a specific auxotroph. For semi-specific auxotrophs, reactions can remain in the model capable of converting close derivative metabolites to the necessary biomass component. This leaves a small set of metabolites which, when supplemented into the media, will individually allow the strain to grow (**Figure B**). The strain designs that have been validated in *in vivo* studies also are shown in **Table A**.

**Major Subsystem Elimination Design**s

MSE designs represent novel results from this model-driven study and account for strains in which a large set of alternative metabolites that can be taken up by the strain to synthesize all of the necessary biomass components blocked by the reaction knockouts. For example, the phosphoenolpyruvate carboxylase (PPC), malate synthase (MALS) and fumarase (FUM) knockout shown in **Fig 3B** has impaired TCA functionality. There are 18 metabolites capable of restoring growth to this knockout strain (**S2 Data**), with many of these being TCA cycle metabolites. When these three reactions are knocked out, four of the TCA cycle metabolites shown in the figure (L-malate, citrate, 2-oxoglutarate, or L-asparagine) can restore growth to the strain if supplemented into glucose minimal media (**Fig 3B**). There, however, are multiple different sets of reactions that can be knocked out to impair TCA cycle functionality. Depending on the specific set of TCA cycle reaction knockouts, the set of restorative metabolites will be altered (**Fig 3B**). This is a consequence of the biosynthetic precursor metabolites that cannot be synthesized by the TCA cycle upon knocking out the reactions, along with the network connectivity and directionality of the reactions remaining in the network.

An MSE design typically impairs a major cellular subsystem itself or the entry of a key precursor metabolite into a major biosynthetic pathway (**Figure B**). As summarized in **S2 Data,** the major subsystems often impaired by OptAux solutions include the citric acid cycle, the pentose phosphate pathway, nucleotide biosynthesis, glycolysis, among others. Furthermore, in some cases, an additional reaction knockout was included by OptAux to increase the uptake requirement of the restorative metabolites of an MSE design. An example of this was the addition of a phosphoserine transaminase (PSERT) knockout to a FUM and PPC knockout strain to further inhibit glycine and serine metabolism. This effectively increased the average uptake requirement of the restorative metabolites by 35% (**S2 Data**) .

# Mutations Targeting Stress Response Functions

Mutations were observed that appeared to augment general stress responses possibly induced by newly introduced auxotrophies. This included mutations acquired in multiple Δ*gltA*Δ*prpC* endpoint clones and one Δ*pyrC* endpoint clone in a host factor protein related to nonspecific global stress responses, *hfq* (**Figure G**). Most of the mutations resulted in amino acid substitutions in the first half of the protein with one observed mutations resulting in a premature stop codon, though the latter case was not seen in any sequenced endpoint clones. This suggests that these mutations act to disable its functionality or augment its behavior. Given that Hfq is an RNA-binding protein involved in numerous cellular functions, the specific effect of these mutations is not clear. Of these functions, however, *hfq* is required for the translation of the S sigma factor [1] and for the regulatory activity of small regulatory RNAs [2]. Therefore these mutations could possibly act to alter the activity of these small RNAs or the activity of global stress response genes regulated by *rpoS* expression.

Beyond augmenting nonspecific stress responses, three different mutations were acquired in all Δ*pyrC* endpoint clones of the Δ*hisD* & Δ*pyrC* co-culture in the *envZ* ORF. This gene is part of the EnvZ/OmpR two-component regulatory system that regulates osmotic response functions in the presence of osmotic stress. One of the three mutations resulted in an amino acid substitution in position 11 which occurs 5 amino acids before the start of a transmembrane protein region [3] with another mutation consisting of a 12 base pair insertion in a beta strand within the periplasmic region [4]. The third mutation consisted of an amino acid substitution at position 241 within an alpha helix of the histidine kinase domain of the protein [5] (**Figure G**). This is also two amino acids down from the location of the L-histidine that is autophosphorylated in response to osmotic stress. These mutations could function to either increase or decrease the expression of the osmotic response functions regulated by OmpR in its phosphorylated state, which largely consists of outer membrane proteins, including transporters [6].

Similarly, mutations were observed that could affect the global cell response to nutrient limitation. This included a mutation in the *sspA* ORF in the Δ*hisD* strain from Δ*hisD* & Δ*gltA*Δ*prpC* co-cultures. Also observed was a 10 kbp deletion which includes *sspA* and *sspB* in a Δ*hisD* endpoint clone of the same co-culture. Intergenic mutations were also seen upstream of *sspA* in the Δ*hisD* strain of Δ*hisD* & Δ*pyrC* co-culture (**Tables E, G**). This protein is induced by low amino acid concentrations and its expression increases with low growth rates [7], both of which would be experienced by the Δ*hisD* strain strain in these co-cultures. Expression of this protein activates P1 bacteriophage expression and activates processes to better survive starvation and certain stresses, such as acid stress [8,9]. Preventing the allocation of cellular resources to these processes would likely provide a growth advantage. Additionally, it has been shown that *hisM*, a subunit of the L-histidine ABC uptake system, is upregulated in *sspA* mutants which could help enhance L-histidine uptake in the Δ*hisD* strain [10].

Both Hfq and EnvZ play a role in regulating outer membrane porin *ompF*. Hfq is required for small RNA MicF repression [11] and expression of RpoS [6,12], of which *ompF* is part of the sigmulon [13]. Alternatively, *ompF* is regulated as part of the osmotic stress response, mediated

by EnvZ [6]. In addition to possible changes is *ompF* activity by altered Hfq and EnvZ activity, two different mutations were observed upstream of *ompF* in Δ*hisD* endpoint clones in two Δ*hisD* & Δ*gltA*Δ*prpC* and all three Δ*hisD* & Δ*pyrC* lineages (**Figure G**). Both of these were SNPs that occurred within 20 base pairs of the transcription start site. Given the complex regulatory relationship among all of these genes, it is difficult to predict what the function of these mutations accomplish beyond possible global stress response mitigation.

# Broad Genome Duplications

There were cases where it was not possible to hypothesize the evolutionary benefit of amplifying a particular duplicated region. This was the case for two consistently duplicated regions. The first of these broadly appeared between the 2,000 kbp - 3,000 kbp genome position and was observed in all three co-culture pairings. Further, this broad duplication or a smaller subset appeared above the 1.25 multiplicity cutoff in at least one population sample in five ALE lineages, though the multiplicity may be understated due to skewing of the strain abundances in co-culture. This is particularly the case for *ΔhisD* & *ΔpyrC* co-cultures where the *ΔpyrC* strain outnumbers the *ΔhisD* strain ~3 fold. Within all 5 lineages with large duplications in this region, the genome region encoding the HisJMPQ histidine ABC uptake complex [14] is duplicated despite the start and end position of this duplication region varying markedly across different ALE lineages (**Figures H-J**). Further, by the end of ALE #11, this initial broad duplication either acquired an additional nested duplication precisely at a 32 gene region containing *hisJMPQ* or a subpopulation with a high multiplicity, targeted duplication in this region emerged (**Fig 7**). Additionally, a clear duplication with a multiplicity of 2.5 in a 27 gene region containing the *hisJMPQ* is observed in the endpoint of ALE #2, further suggesting the duplications provide a community benefit by increasing the expression of or encouraging mutations in this transporter, likely in the *ΔhisD* strain (**Figure J**), to improve L-histidine uptake.

Similar to *hisJMPQ*, broad duplication regions were observed containing *dctA,* which is mutated in Δ*pyrC* strains in co-culture and codes for a proton symporter that transports citrate, orotate, or C4-carboxylic acids [15]. These duplicated regions appeared in all three Δ*hisD* & Δ*pyrC* co-cultures approximately between 3,600 kbp - 3,700 kbp with a multiplicity ranging from 1.5 to 3 (**Figure J**). It is possible this duplication functions, in addition to the mutations upstream of *dctA* (**Table G**), to further amplify the expression of or encourage mutations in this transporter. Considering that, of the possible metabolites imported by DctA, only orotate is computationally predicted to be able to restore growth in a Δ*pyrC* mutant, this further hints that Δ*pyrC* is being cross-fed orotate in the evolved communities.

# Duplications in Endpoint Clones

Though stark duplications peaks appeared in many populations, most were not observed in the endpoint clones (**Figures H-J**). The only clones containing duplications included three Δ*hisD* endpoint clones in the Δ*hisD* & Δ*pyrC* co-culture and consisted of a narrow region containing *hisJMPQ*, similar to those described in the population samples. It is unclear why many of the endpoint clones did not possess duplications, but there a few possible explanations. First, the

duplication could have been reversed *in vivo* over the course of the ~25 cell divisions required, when starting with an isolated clone, to produce the amount of DNA needed to perform genome sequencing. Genome duplications are inherently unstable and are detrimental to cellular fitness if they provide no additional growth benefit, as would be the case when the auxotrophs are growing in rich media. Thus duplications can be reversed rather quickly by homologous recombination at a rate as high as 0.15 per cell per generation [16,17]. Alternatively, it is possible that the duplications observed in the populations could have been a result of high multiplicity duplications in small subpopulations within the co-cultures. It is possible that these cells were simply not isolated by chance.

## Model Computed Metabolic Cross-Feeding

An important community feature is the metabolic cross-feeding occurring between strains in co-culture. This can be assessed by observing the metabolite exchange between strains in simulation, with varying fractional strain abundances. The computed metabolic cross-feeding for the substrate and proteome limited ME-model simulations provide markedly different predictions (**Figure M**). The substrate limited simulations provide relatively clear predictions of metabolic cross-feeding, assuming that the co-culture communities evolve to cross-feed only one metabolite, which is supported by the mutational analysis. Further, two of the three dominate cross-feeding metabolites in the simulations confer with the experimentally inferred cross-feeding metabolites from the mutation data (orotate for Δ*hisD* & Δ*pyrC* and 2-oxoglutarate for Δ*hisD* & Δ*glta*Δ*prpC,* **Table 2**). Alternatively, for two of the three co-cultures, no clear prediction of the cross-fed metabolites can be inferred from the proteome limited simulations. The identity of metabolites cross-fed in community, however, is likely largely dependent on model parameters such as enzyme turnover rates. Given the existence of alternate optimal solutions inherent in M-model simulations, M-model metabolite cross-feeding is not shown.

# Supplementary Figures

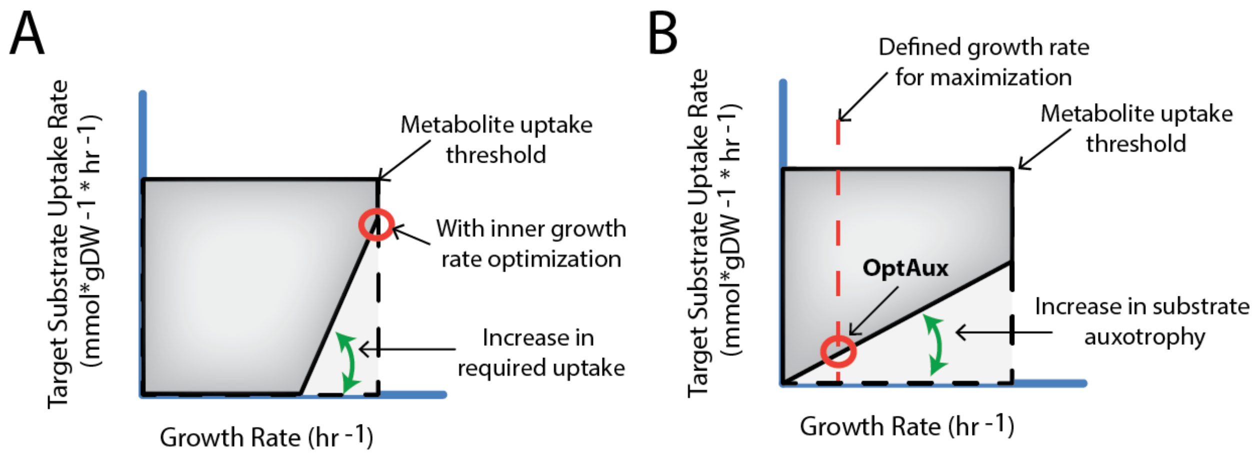


**Figure A. Solutions for existing MILP algorithms versus OptAux solutions.** (**A**) Existing strain design MILP algorithms (e.g. OptKnock with tilting [18] and RobustKnock [19]), if implemented to optimize metabolite uptake, will give solutions similar to this. This is due to an inner growth rate optimization that causes the point circled in red to be optimized. This causes no uptake of the metabolite to be required at low growth rates, however, which is undesirable. (**B**) For OptAux, the inner growth rate optimization is replaced with a parameter that defines the growth rate in which the optimization will be performed. If this parameter is close to zero, OptAux will prefer solutions similar to what is shown. This is desirable because the metabolite is required at all simulated growth rates.

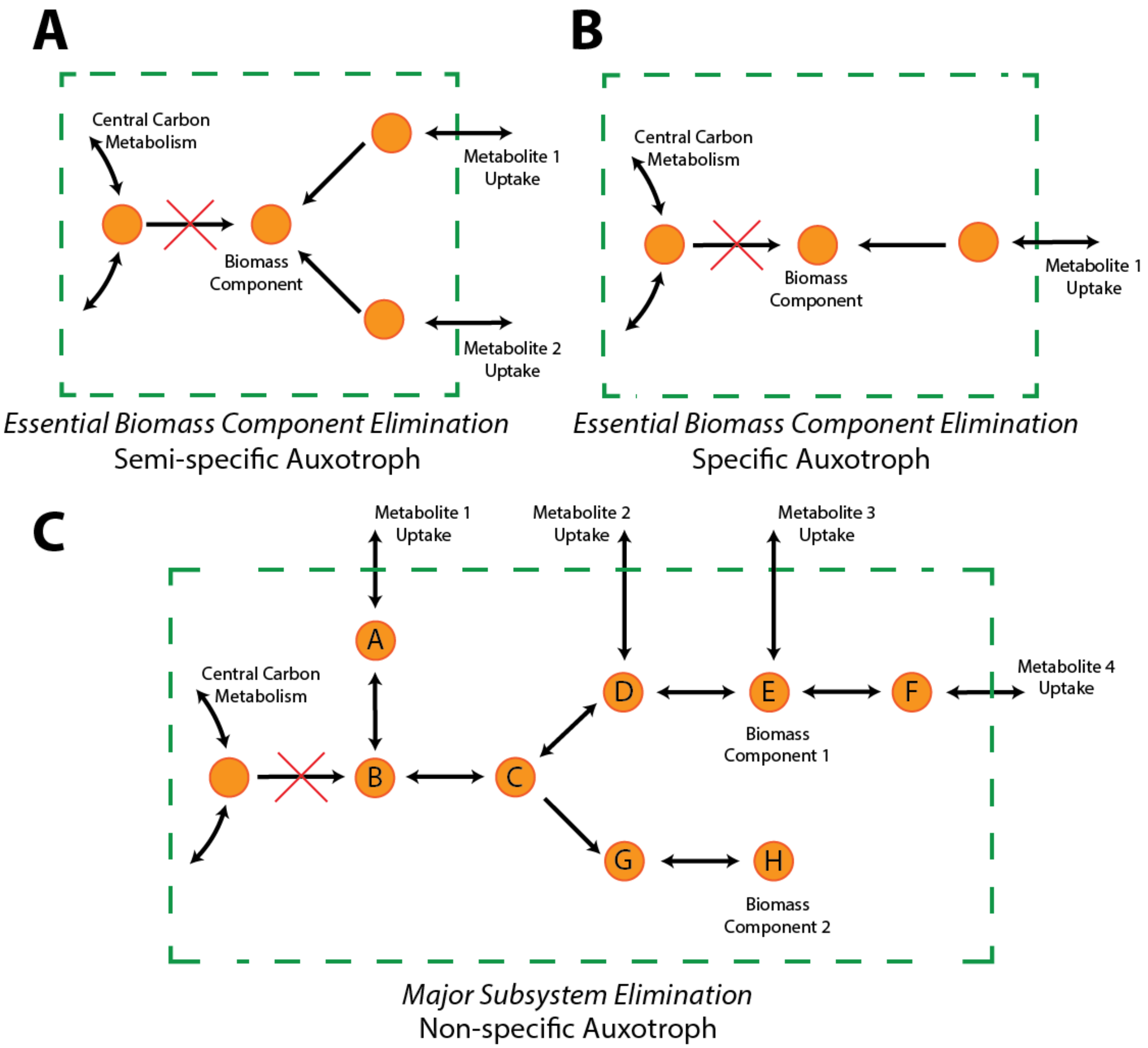


**Figure B. Categories of OptAux solutions**. **A**: Semi-specific auxotrophs can grow when one of a small set of metabolites is present. Typically, these metabolites are closely related to each other chemically. As shown, this can represent cases where multiple metabolites can be interconverted into a component of the biomass objective function. **B**: Specific auxotrophs only can grow in the presence of exactly one metabolite. **C**: Major subsystem elimination auxotrophs can grow in the presence of many different metabolites. As shown above, metabolites 1, 2, 3, and 4 can all be interconverted into biomass component 1 and 2. In the case of metabolite 1 this can be accomplished through pathway **A → B → C → D → E** along with **A → B → C → G → H**.

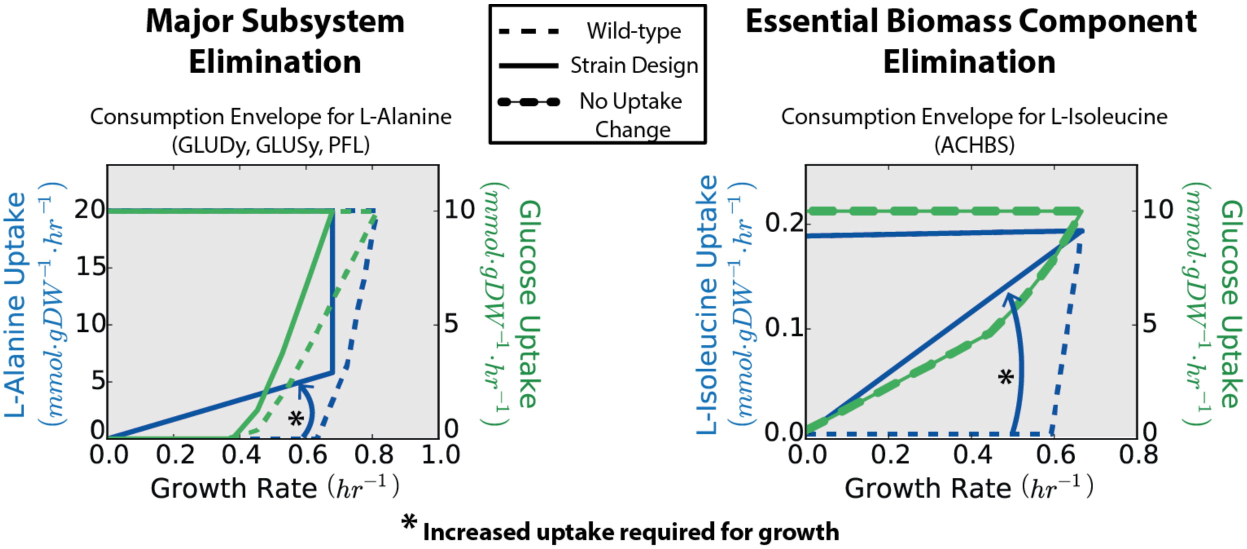


**Figure C. Metabolite uptake requirement for the two OptAux solution types.** The different uptake requirements are shown through two consumption envelopes where the bottom and top lines represent the minimum and maximum possible uptake, respectively, of the substrate at each growth rate. Major subsystem elimination solutions are nonspecific in regards to which metabolites can be supplemented into the media in order for the strain to grow. A consequence of this, however, is that they require significant uptake of whatever metabolite is supplemented. For the major subsystem elimination example shown, the strain is unable to utilize glucose as efficiently with the triple knockout shown in parenthesis. Therefore, it requires large amounts of L-alanine to synthesize the biomass precursors unable to be synthesized by glucose, and the required glucose uptake is altered relative to the dashed wild-type line. For the essential biomass component elimination, L-isoleucine is the only biomass precursor unable to be synthesized by glucose. Therefore, L-isoleucine is required in low amounts and glucose uptake to unchanged.

**Table A. Summary of Essential Biomass Component Elimination knockouts.**
The table lists all unique specific auxotrophs predicted by OptAux. These designs are predicted to only grow only when the media is supplemented with the listed metabolite. If these reaction knockouts have been used experimentally, then the study is listed as well. A KDO(2)-lipid IV(A) auxotroph was also found with OptAux, but was excluded from this table as it is likely a model artifact.

| Reaction Knockout | Auxotrophs | Study, if knockout characterized | Validated? |
|---|---|---|---|
| **Specific Auxotrophs (no substitute metabolite can restore growth)** | | | |
| MOHMT (panB) | (R)-Pantothenate | Validated in [20]. Can grow slightly with pantoate or ketopantoate | Yes |
| DHQTi (aroD) | Shikimate | No citation confirming auxotrophy. | New Prediction |
| AMPMS2 (thiC) | Thiamin | Computationally equivalent to TYRL (thiG), validated in [21] | Yes |
| ACLS ((ilvN and ilvB) or (ilvI and ilvH)) | L-Valine | OptAux solutions have same GPR. | No |
| ACHBS ((ilvN and ilvB) or (ilvI and ilvH)) | L-Isoleucine | | |
| IPPS (leuA) | L-Leucine | Computationally equivalent to IPMD (leuB) used in [22] and further validated in [23]. | Yes |
| OCBT (argF and argI) | L-Arginine | Validated in [24]. Computationally equivalent to ARGSL (ArgH) KO validated in [25] | Yes |
| PPNDH (pheA) | L-Phenylalanine | Used in [22] | Yes |
| IGPDH (hisB) | L-Histidine | Used in [22] | Yes |
| HSK (thrB) & PTHRpp (aphA) | L-Threonine | HSK knockout computationally equivalent to THRS (thrC) used in [26]. Computationally necessary to knockout PTHRpp to prevent growth on L-threonine O-3-phosphate. | Yes |
| HSST (metA) & TRDR ((trxA and trxB) or (trxB and trxC)) | L-Methionine | HSST (metA) knockout used in [22] | Yes |
| ASNS2 (asnA) & ASNS1 (asnB) | L-Asparagine | Validated in [27] | Yes |
| P5CR (proC) & AMPTASEPG (pepA or pepD or pepB or pepN) | L-Proline | No citations. G5SD (ProA) used in [22] though this is not computationally predicted to be auxotrophic. | New Prediction |
| PPND (tyrA) & TYRPpp (aphA) | L-Tyrosine | PPND (tyrA) knockout used in [22] | Yes |
| ALLTN (allB) & DBTS (bioD) | Biotin | DBTS (bioD) confirmed in [26,28]. ALLTN knockout unnecessary | Yes |
| CYSS (cysK or cysM) & AMPTASECG (pepD or pepN or pepA or pepB) | L-Cysteine | Computationally equivalent to SERAT (cysE) used in [22]AMPTASECG to prevent growth on reduced glutathione and L-cysteinylglycine | Yes |
| ASNN (ansA or iaaA) & ASNNpp (ansB) & ASPTA (aspC) | L-Aspartate | No citations. tyrB is an isozyme of ASTPA but is not in iJO1366 GPR*s* | No, *aspC* is multifunctional |
| GLUDy (gdhA) & GLUSy (gltB and gltD) & ALAR (dadX or alr) | L-alanine-D-glutamate-meso-2,6-diaminoheptanedioate-D-alanine | No citations | New Prediction |

| | | | |
|---|---|---|---|
| ASPCT ((pyrI and pyrB) or pyrB) & UPPRT (upp) & URIK2 (udk) \| URIK2 (udk) & DHORTS (pyrC) & UPPRT (upp) | Orotate | No citations | New Prediction |

**Table B. Summary of auxotroph mutants used for co-culture ALEs**

| Mutant | Δ*hisD* | Δ*gdhA*Δ*gltB* | Δ*gltA*Δ*prpC* | Δ*pyrC* |
|---|---|---|---|---|
| **OptAux Solution Type** | Essential biomass component elimination | Major subsystem elimination | Major subsystem elimination | Major subsystem elimination |
| **Reaction(s) Disabled** | Histidinol dehydrogenase (HISTD) | Glutamate synthase (GLUSy), Glutamate dehydrogenase (GLUDy) | Citrate Synthase (CS) | Dihydroorotase (DHORTS) |
| **Process Disabled** | Histidine Synthesis | Nitrogen Assimilation in Amino Acid Synthesis | Citric Acid Cycle | Purine and Pyrimidine Biosynthesis |

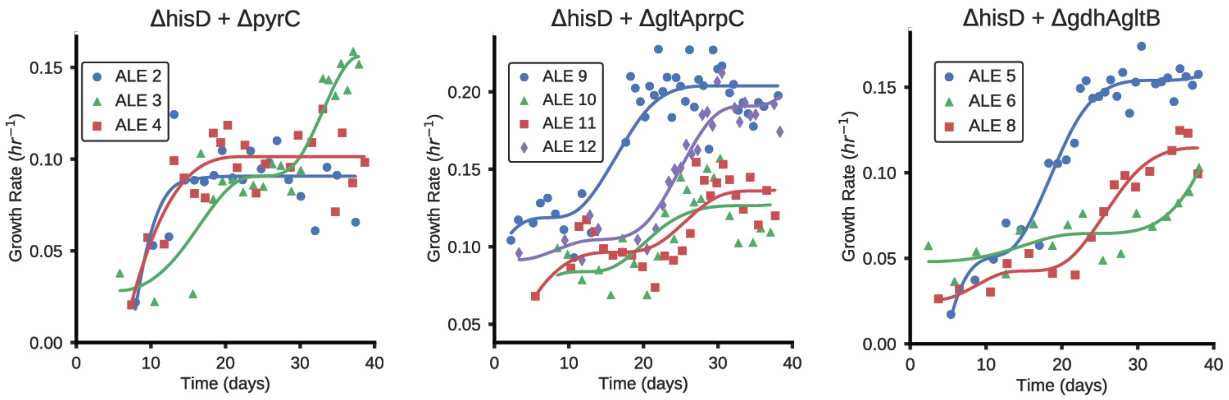


**Figure D**. **ALE growth trajectories of all replicates for the three co-culture pairs.**

**Table C. Characteristic mutations observed in each starting strain prior to co-culture evolution**

| | **Mutant** | **Δ*hisD*** | **Δ*gdhA*Δ*gltB*** | **Δ*prpC*Δ*gltA*** | **Δ*pyrC*** |
|---|---|---|---|---|---|
| **Characteristic Mutation 1** | **Gene** | **lrhA / alaA** | **yjiC** | **kgtP/ rrfG** | **cyoB** |
| | **Mutation** | MOB (IS5 (+) +4 bp) | G→T | T→C | C→T |
| | **Protein Change** | intergenic (- 104/- 816) | H18Q (CAC→CAA) | intergenic (- 321/+2) | W440* (TGG→TAG) |
| | **Gene Function** | DNA-binding transcriptional regulator for motility functions / glutamate-pyruvate aminotransferase | Putative Protein | Akg symporter / 5S ribosomal RNA | Cytochrome bo oxidase |
| **Characteristic Mutation 2** | **Gene** | **yqiC** | **yqiC** | - | - |
| | **Mutation** | MOB (IS5 (+) +4 bp) | G→T | | |
| | **Protein Change** | coding (56- 59/291 nt) | E27* (GAA→TAA) | | |
| | **Gene Function** | Conserved protein (stress induced mutagenesis response) | Conserved protein (stress induced mutagenesis response) | | |

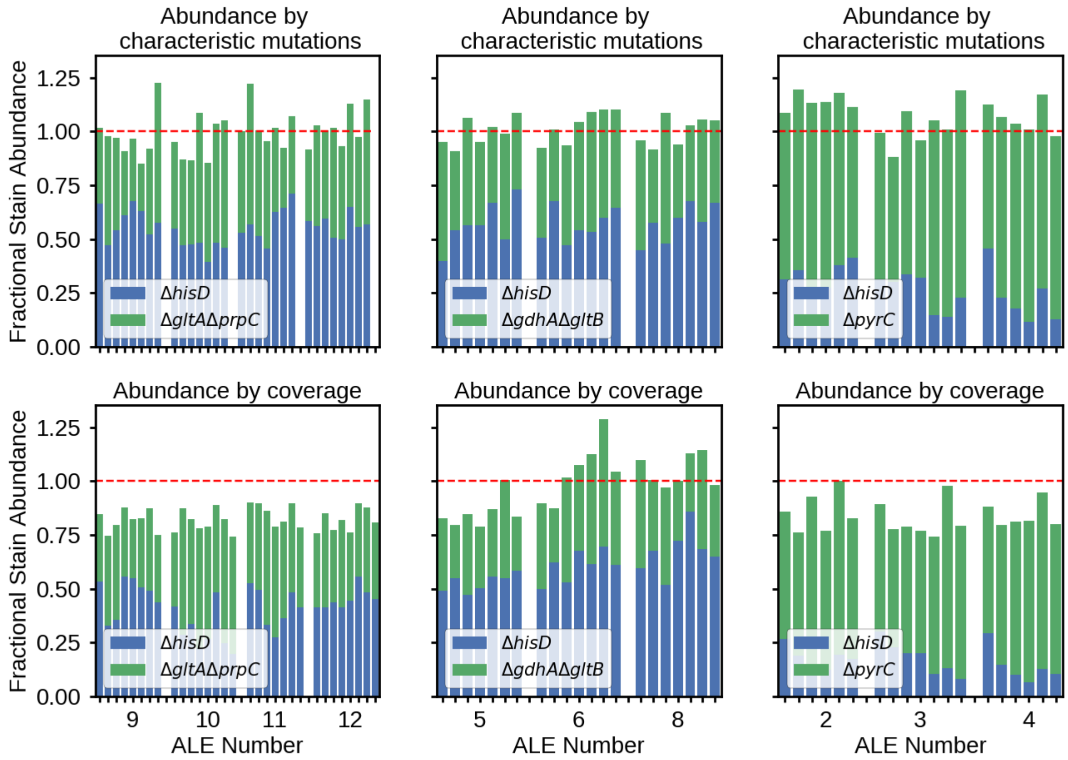


**Figure E. Relative abundance predictions.** Fractional abundances in the top row are predicted from the frequency of characteristic mutations. The bottom row is based on the coverage of the knocked out genes relative to the average read coverage across the genome. Each bar represents the experimentally inferred fractional abundance of the corresponding strain in community. Since the inferred abundances rely on the observed frequency of a particular mutation or sequencing read depth, there will be some inherent stochasticity in the measurements and therefore the predictions. Given this stochasticity, the fractional strain abundances will likely not sum exactly to 100%, but, to have confidence in the predictions, it should sum close to 100%. This is shown to be the case for the characteristic mutation-based computation. For the coverage-based computation, the total predicted relative abundances in cases sum to below 100%, likely due to duplications causing an increase in the coverage fit mean provided by the sequencing software.

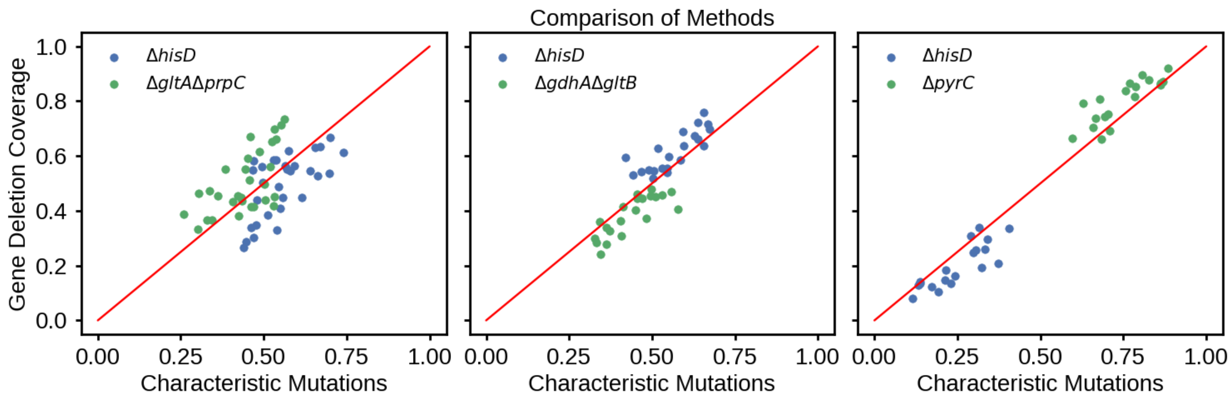


**Figure F**. **Comparison of coverage and characteristic mutation based predictions of community composition.** Shown after normalizing by the sum of predicted fractions.

**Table D.** Gene and reactions knockouts for each of the four strains used in the co-culture ALEs. The metabolites that are blocked in the biomass objective function due to the knockouts are listed as well as all computationally predicted metabolites that can individually restore growth in the strain when supplemented in the media (i.e., auxotrophic metabolites).

| **Reaction Knockouts** | **Gene Knockouts** | **Gene Knockout Names** | **Blocked Biomass compounds in the Biomass Objective Function (BIGG)** | **Auxotrophic Metabolites (BIGG)** |
|---|---|---|---|---|
| GLUDy & GLUSy | b1761 & b3212 and b3213 | gdhA & gltB and gltD | cys__L_c, pydx5p_c, pe161_p, his__L_c, glu__L_c, utp_c, pe160_p, pe161_c, thmpp_c, dctp_c, pe160_c, ribflv_c, atp_c, murein5px4p_p, datp_c, pro__L_c, ser__L_c, nad_c, coa_c, thf_c, ctp_c, thr__L_c, val__L_c, gly_c, bmocogdp_c, leu__L_c, lys__L_c, phe__L_c, ala__L_c, ile__L_c, nadp_c, met__L_c, tyr__L_c, amet_c, dttp_c, gln__L_c, btn_c, mlthf_c, asp__L_c, asn__L_c, dgtp_c, pheme_c, 10fthf_c, sheme_c, gtp_c, arg__L_c, fad_c | EX_4abut_e, EX_LalaDglu_e, EX_LalaDgluMdap_e, EX_LalaDgluMdapDala_e, EX_LalaLglu_e, EX_agm_e, EX_ala__D_e, EX_ala__L_e, EX_alaala_e, EX_arg__L_e, EX_asn__L_e, EX_asp__L_e, EX_gln__L_e, EX_glu__L_e, EX_gthrd_e, EX_orn_e, EX_pro__L_e, EX_progly_e, EX_ptrc_e |
| CS | b0720 | gltA | glu__L_c, murein5px4p_p, pro__L_c, thf_c, gln__L_c, mlthf_c, pheme_c, 10fthf_c, sheme_c, arg__L_c | EX_LalaDglu_e, EX_LalaDgluMdap_e, EX_LalaDgluMdapDala_e, EX_LalaLglu_e, EX_akg_e, EX_arg__L_e, EX_cit_e, EX_fe3dcit_e, EX_gln__L_e, EX_glu__L_e, EX_gthrd_e, EX_orn_e, EX_pro__L_e, EX_progly_e |
| HISTD | b2020 | hisD | his__L_c | EX_his__L_e |
| DHORTS | b1062 | pyrC | utp_c, dctp_c, ctp_c, dttp_c | EX_23ccmp_e, EX_23cump_e, EX_3cmp_e, EX_3ump_e, EX_cmp_e, EX_csn_e, EX_cytd_e, EX_dcmp_e, EX_dcyt_e, EX_dump_e, EX_duri_e, EX_orot_e, EX_uacgam_e, EX_udpacgal_e, EX_udpg_e, EX_udpgal_e, EX_udpglcur_e, EX_ump_e, EX_ura_e, EX_uri_e |

**Table E.** Mutations in the endpoint clones of the Δ*hisD* & Δ*gltA*Δ*prpC* co-culture

| Strain w/ Mutation | Gene | Specific Function | Mutation | Protein Change | Ale Numbers |
|---|---|---|---|---|---|
| **Δ*hisD* & Δ*gltA*Δ*prpC* Mutations** | | | | | |
| **Δ*hisD*** | *hisJ / argT* | histidine ABC transporter periplasmic binding protein | SNP | intergenic (- 86/+135) | 9 |
| | | | SNP | intergenic (- 127/+94) | 9 |
| | | | SNP | intergenic (- 86/+135) | 10 |
| | | | SNP | intergenic (- 61/+160) | 12 |
| | | | INS | intergenic (- 69/+152) | 12 |
| | *[yhcE], yhcF, yhcG, yhcH, nanK, nanE, nanT, nanA, nanR, dcuD, sspB, sspA* | **yhcFGH:** DUF1016 domain-containing proteins **nanKETAR:** N-acetylneuraminate uptake and metabolism proteins **dcuD**: putative C4-dicarboxylate uptake C family transporter **sspAB**: Stringent starvation proteins | DEL | IS5- mediated | 9 |
| | *ompF / asnS* | outer membrane porin 1a (Ia;b;F) / asparaginyl tRNA synthetase | SNP | intergenic (- 127/+476) | 9 |
| | | | SNP | intergenic (- 122/+481) | 12 |
| | *sspA* | stringent starvation protein A | SNP | W166* (TGG→TGA) | 12 |
| | *glnK* | nitrogen assimilation regulatory protein for GlnL, GlnE, and AmtB | SNP | K79* (AAG→TAG) | 10 |
| **Δ*gltA*Δ*prpC*** | | | DEL | coding (40/339 nt) | 9 |
| | | | DEL | coding (91/339 nt) | 11 |
| | | | DEL | coding (73/339 nt) | 12 |
| | *kgtP* | alpha-ketoglutarate transporter | SNP | P124Q (CCG→CAG) | 9 |
| | | | SNP | G143A (GGC→GCC) | 10 |
| | *hfq* | global sRNA chaperone; HF-I, host factor for RNA phage Q beta replication | SNP | I44T (ATC→ACC) | 9 |
| | | | SNP | G34V (GGG→GTG) | 10 |
| | | | SNP | I44N (ATC→AAC) | 12 |

| | | | | | |
|---|---|---|---|---|---|
| | *iap* | aminopeptidase in alkaline phosphatase isozyme conversion | SNP | G134S (GGC→AGC) | 10 |
| | *nlpD* | activator of AmiC murein hydrolase activity, lipoprotein | SNP | Q66* (CAG→TAG) | 10 |
| | *prfB* | peptide chain release factor RF-2 | SNP | A188T (GCT→ACT) | 10 |
| | *sdhA* | succinate dehydrogenase, flavoprotein subunit | SNP | E551* (GAA→TAA) | 10 |
| | *guaD* | guanine deaminase | SNP | M169I (ATG→ATT) | 10 |
| | *rpoS* | RpoS stabilzer during Mg starvation, anti-RssB factor | DEL | coding (482/993 nt) | 11 |
| | *ygeG* | SycD-like chaperone family TPR-repeat-containing protein | SNP | D126Y (GAT→TAT) | 11 |

**Table F**. Mutations in the endpoint clones of the Δ*hisD* & Δ*gdhA*Δ*gltB* co-culture

| Strain w/ Mutation | Gene | Specific Function | Mutation | Protein Change | Ale Numbers |
|---|---|---|---|---|---|
| **Δ*hisD* & Δ*gdhA*Δ*gltB* Mutations** | | | | | |
| **Δ*hisD*** | *hisJ / argT* | histidine ABC transporter periplasmic binding protein / lysine/arginine/ornithine transporter subunit | SUB | intergenic (- 61/+159) | 5 |
| | | | INS | intergenic (- 71/+150) | 5 |
| | | | SNP | intergenic (- 86/+135) | 6 |
| | | | SNP | intergenic (- 86/+135) | 8 |
| | *hisJ* | histidine ABC transporter periplasmic binding protein | SNP | D183G (GAT→GGT) | 5,8 |
| | *wecE* | TDP-4-oxo-6-deoxy-D-glucose transaminase | SNP | E278* (GAG→TAG) | 5 |
| | *yifN* | PemK toxin family pseudogene | DEL | pseudogene (178/243 nt) | 5 |
| | *glnA* | glutamine synthetase | SNP | V186E (GTA→GAA) | 6 |
| | *ygjI* | putative transporter | SNP | R83G (CGT→GGT) | 8 |
| | *argR* | l-arginine-responsive arginine metabolism regulon transcriptional regulator | INS | coding (47/471 nt) | 8 |
| **Δ*gdhA*Δ*gltB*** | *glnL* | sensory histidine kinase in two-component regulatory system with GlnG | SNP | I12S (ATC→AGC) | 5 |
| | *yhdW* | pseudogene, ABC transporter | INS | pseudogene (59/66 nt) | 5 |
| | *mngB* | alpha-mannosidase | SNP | L306P (CTG→CCG) | 6 |
| | *asnW / yeeO* | tRNA-Asn / putative multdrug exporter, MATE family | SNP | intergenic (- 8/+93) | 6 |
| | *glnG* | fused DNA-binding response regulator in two-component regulatory system with GlnL: response regulator/sigma54 interaction protein | SNP | D86N (GAT→AAT) | 6 |
| | | | SNP | V18E (GTG→GAG) | 8 |
| | *rne* | endoribonuclease; RNA-binding protein; RNAdegradosome binding protein | SNP | D338G (GAC→GGC) | 8 |

**Table G**. Mutations in the endpoint clones of the Δ*hisD* & Δ*pyrC* co-culture

| Strain w/ Mutation | Gene | Specific Function | Mutation | Protein Change | Ale Numbers |
|---|---|---|---|---|---|
| **ΔhisD & ΔpyrC Mutations** | | | | | |
| **Δ*hisD*** | *hisJ / argT* | histidine ABC transporter periplasmic binding protein / lysine/arginine/ornithine transporter subunit | SNP | intergenic (- 86/+135) | 2,3,4 |
| | *ompF / asnS* | outer membrane porin 1a (Ia;b;F) / asparaginyl tRNA synthetase | SNP | intergenic (- 122/+481) | 2,3,4 |
| | *sspA / rpsI* | stringent starvation protein A / 30S ribosomal subunit protein S9 | SNP | intergenic (- 356/+39) | 2,3,4 |
| | *ybdL* | Conserved Protein | SNP | A378V (GCA→GTA) | 2 |
| **Δ*pyrC*** | *dctA / yhjK* | C4-dicarboxylic acid, orotate and citrate transporter / cyclic-di-GMP phosphodiesterase | DEL | intergenic (- 26/+155) | 2 |
| | | | INS | intergenic (- 53/+130) | 3 |
| | | | INS | intergenic (- 64/+119) | 4 |
| | *envZ* | sensory histidine kinase in two-component regulatory system with OmpR | INS | coding (399/1353 nt) | 2 |
| | | | SNP | V241G (GTA→GGA) | 3 |
| | | | SNP | S11P (TCA→CCA) | 4 |
| | *hfq* | global sRNA chaperone; HF-I, host factor for RNA phage Q beta replication | SNP | T61N (ACT→AAT) | 4 |

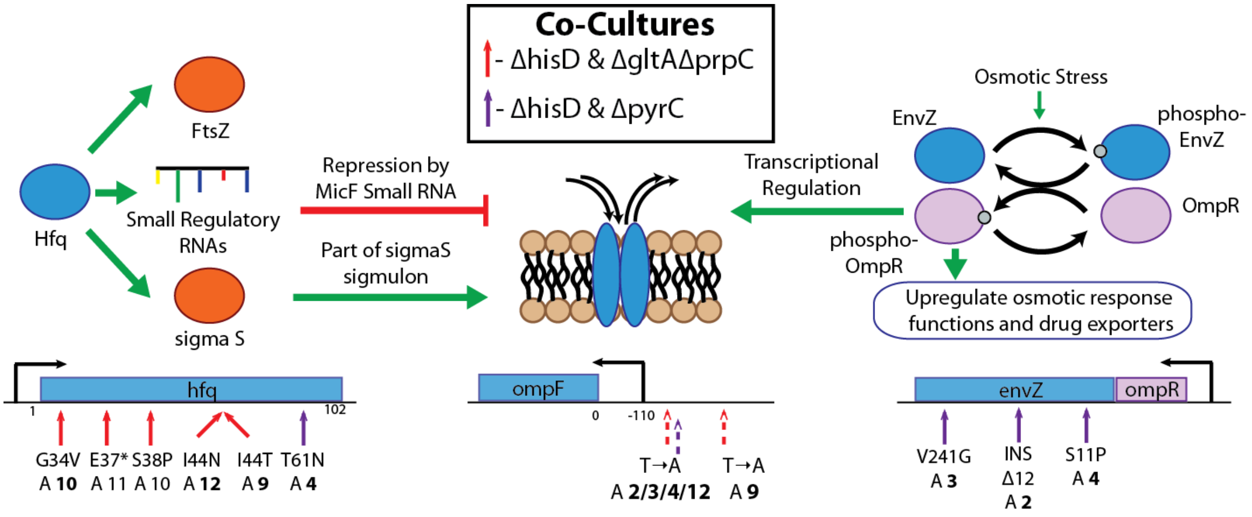


**Figure G**. **Mutations observed associated with the general *E. coli* stress response.** Numerous mutations were observed in the ORF of Hfq which is an RNA-binding protein with numerous global functions. These include interactions with small regulatory RNAs which are often required to enable the small RNA's regulatory function. Hfq is also required for the wild-type expression of the S sigma factor. Both MicF and sigma S are involved in regulating the expression of outer membrane porin *ompF*, a gene which acquired mutations in multiple ALE lineages. Mutations were also observed in the *envZ* ORF which is the sensory protein in the osmotic stress two-component regulatory system. Upon sensing osmotic stress, EnvZ autophosphorylates and transfers a phosphate to OmpR, thus upregulating osmotic stress genes. These genes consist of many outer membrane porins, including *ompF*.

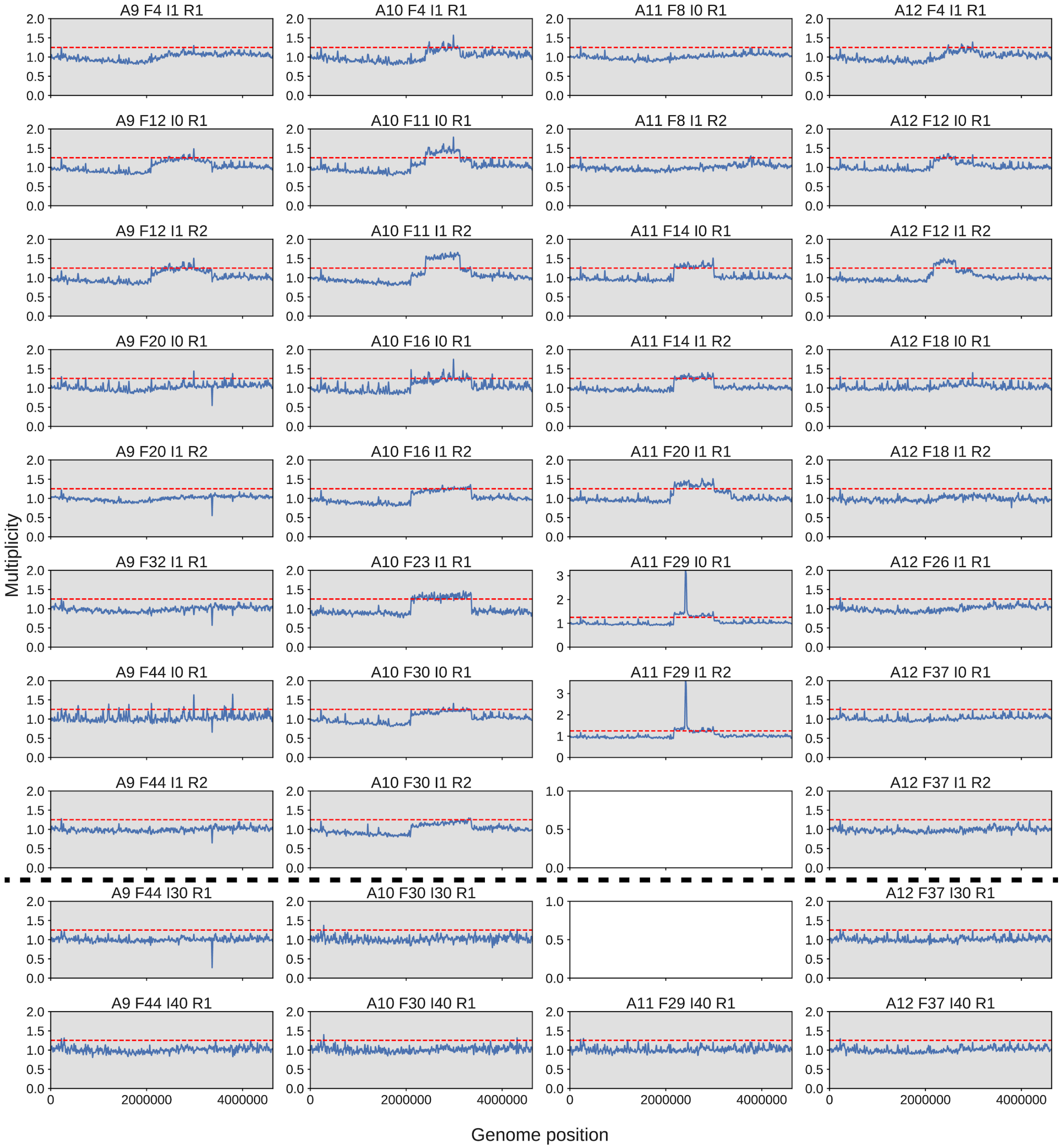


**Figure H**. **Duplications in Δ*hisD* & Δ*gltA*Δ*prpC*.** Dashed red line at 1.25 to denote the duplication cutoff. The population samples and ALE endpoint clones are plotted above and below the dashed line, respectively. Missing plots indicate that the endpoint clone could not be isolated for that strain or that there was an error in sequencing.

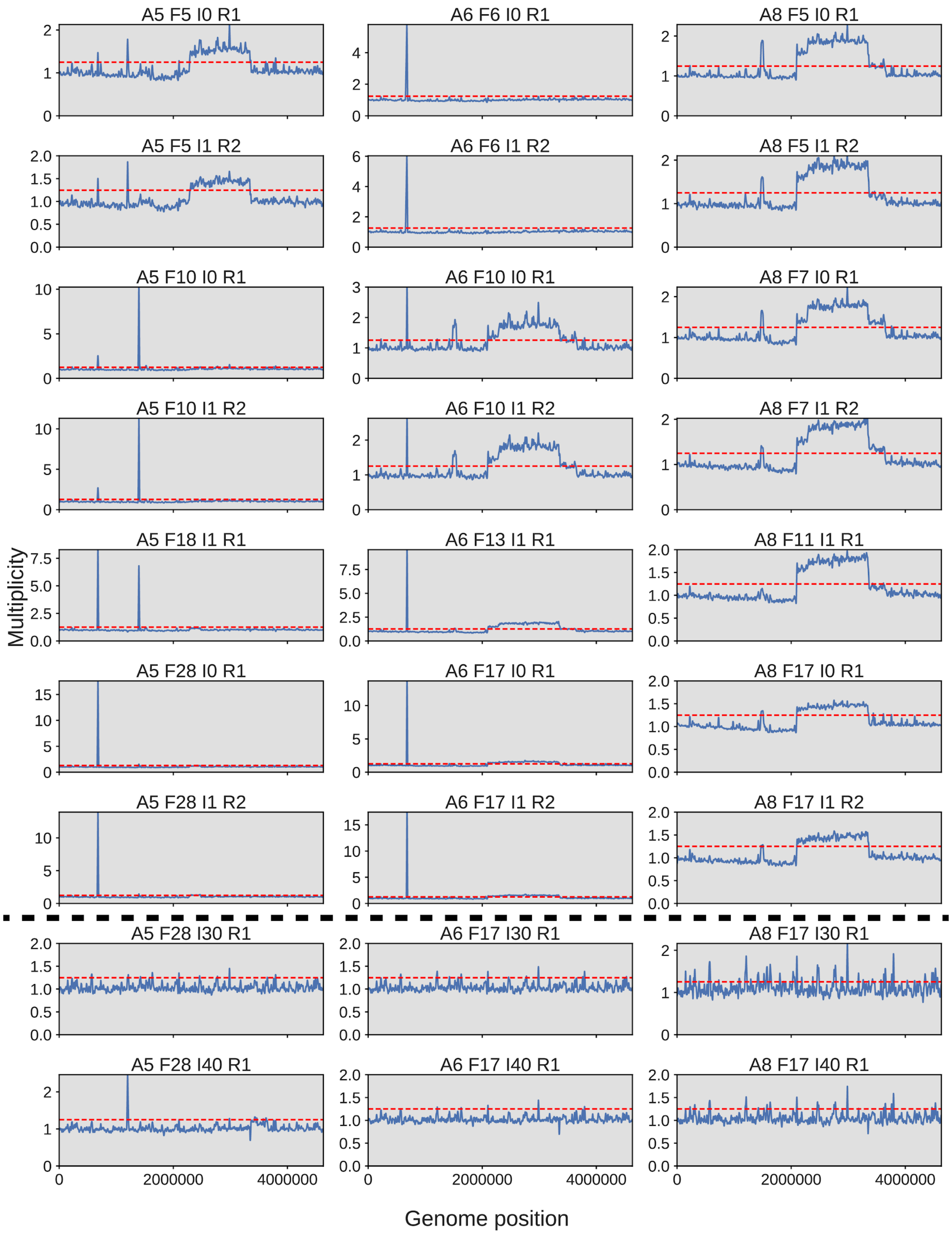


**Figure I. Duplications in Δ*hisD* & Δ*gdh*AΔ*gltB***. Dashed red line at 1.25 to denote the duplication cutoff.The population samples and ALE endpoint clones are plotted above and below the dashed line, respectively.

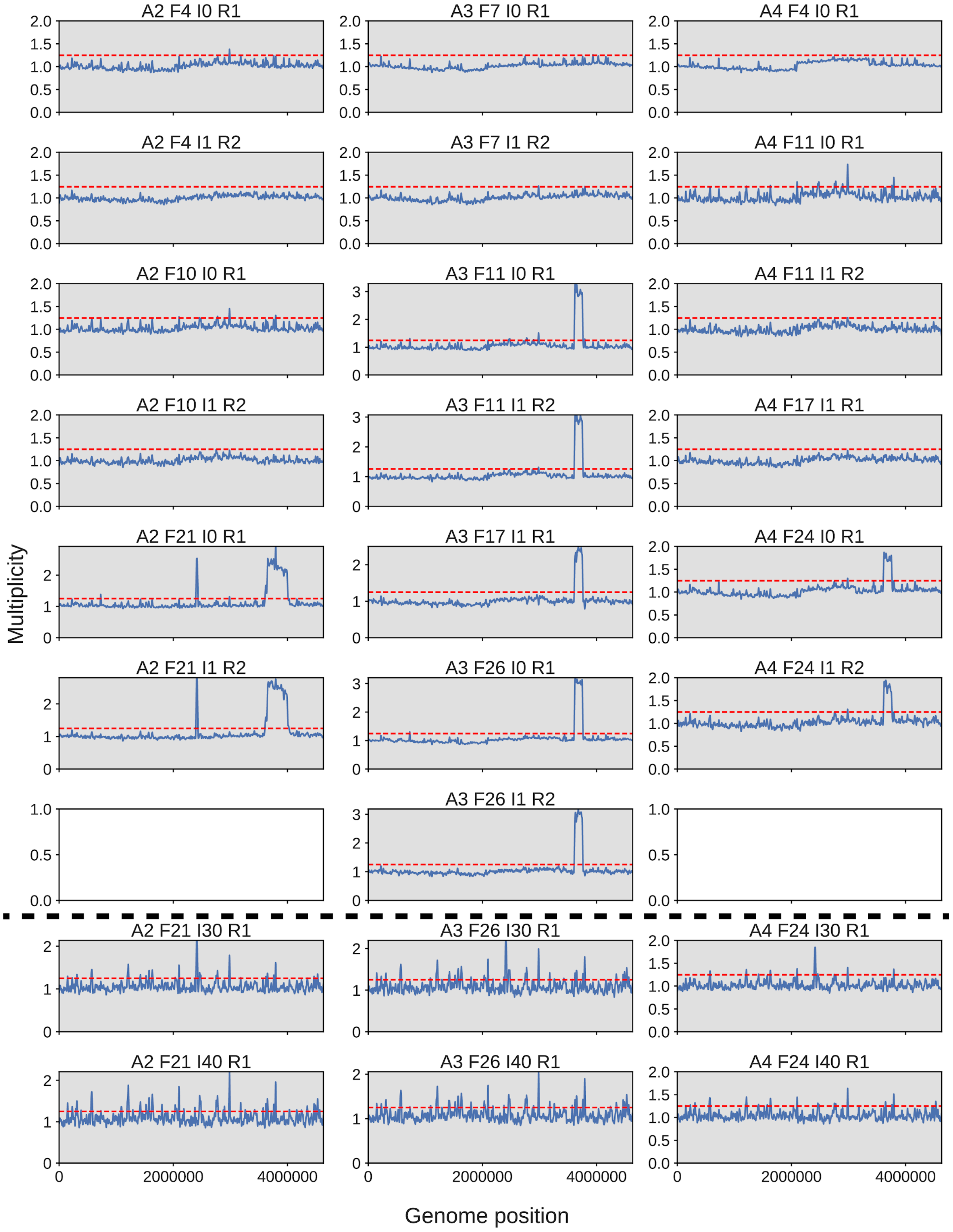


**Figure J. Duplications in Δ*hisD* & Δ*pyrC*.** Dashed red line at 1.25 to denote the duplication cutoff. The population samples and ALE endpoint clones are plotted above and below the dashed line, respectively. Missing plots indicate that there was an error in sequencing.

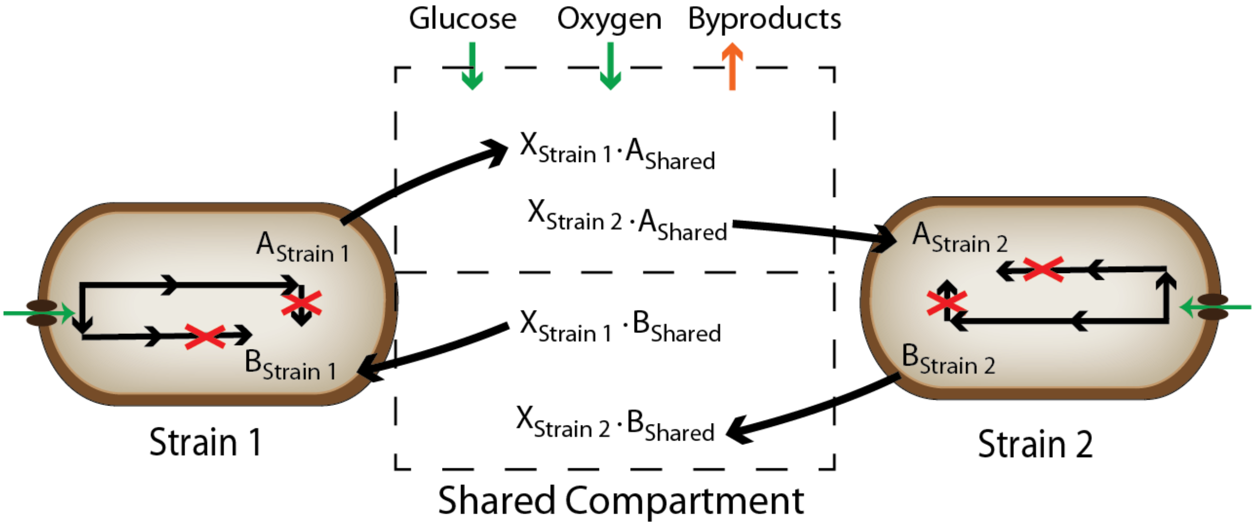


$$\left[\frac{\text{mmol}}{\text{gDW}_{\text{Strain1}}\cdot\text{hr}^{-1}}\right]\cdot\left[\frac{\text{gDW}_{\text{Strain1}}}{\text{gDW}_{\text{Comm}}}\right]=\left[\frac{\text{mmol}}{\text{gDW}_{\text{comm}}\cdot\text{hr}^{-1}}\right]$$

$$V_{\text{Strain 1}}\cdot X_{\text{Strain 1}}=V_{\text{Shared}}$$

$$\left[\frac{\text{mmol}}{\text{gDW}_{\text{Comm}}\cdot\text{hr}^{-1}}\right]\cdot\left[\frac{\text{gDW}_{\text{Comm}}}{\text{gDW}_{\text{Strain2}}}\right]=\left[\frac{\text{mmol}}{\text{gDW}_{\text{Strain2}}\cdot\text{hr}^{-1}}\right]$$

$$V_{\text{Shared}}\cdot\frac{1}{X_{\text{Strain 2}}}=V_{\text{Strain 2}}$$

**Figure K. Community ME-modeling.** Each strain in the community ME-model occupies its own compartment with reaction fluxes in units of mmol $\cdot$ $gDW_{Strain\ i}^{-1}$ $\cdot$ $hr^{-1}$. The strains share a community compartment with reaction fluxes in units of mmol $\cdot$ $gDW_{Community}^{-1}$ $\cdot$ $hr^{-1}$. The metabolites being exchanged in or out of the compartment are multiplied by a $X_{Strain\ i}$ term which corresponds to the fractional abundance of the strain "i" by mass. This term has units of $gDW_{Strain\ i}$ $\cdot$ $gDW_{Community}^{-1}$. The unit conversions are shown below the community modeling depiction.

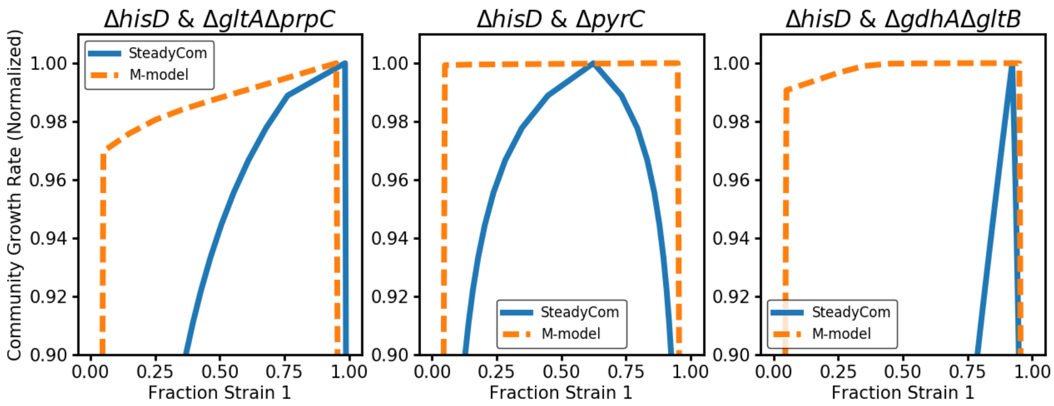


**Figure L. Comparison between SteadyCom and community M-model simulations.**

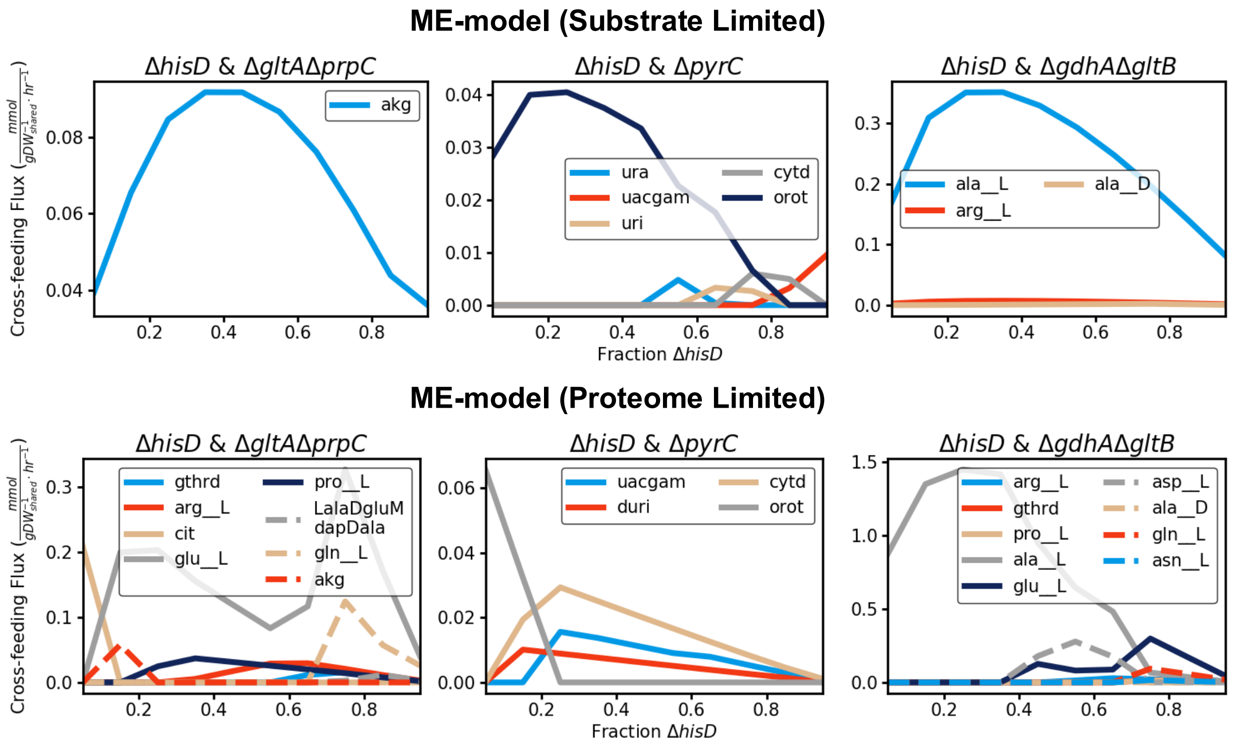


**Figure M. Optimal metabolite cross-feeding of ME-model and M-model simulation.** The community models in these simulations were allowed to cross-feed any metabolite(s) for which the Δ*hisD* partner strain was computationally predicted to be auxotrophic (**Table D**). Positive flux corresponds to cross-feeding from the Δ*hisD* strain to its partner. The L-histidine flux to the Δ*hisD* strain is not shown since it is known that this strain will require L-histidine cross-feeding.

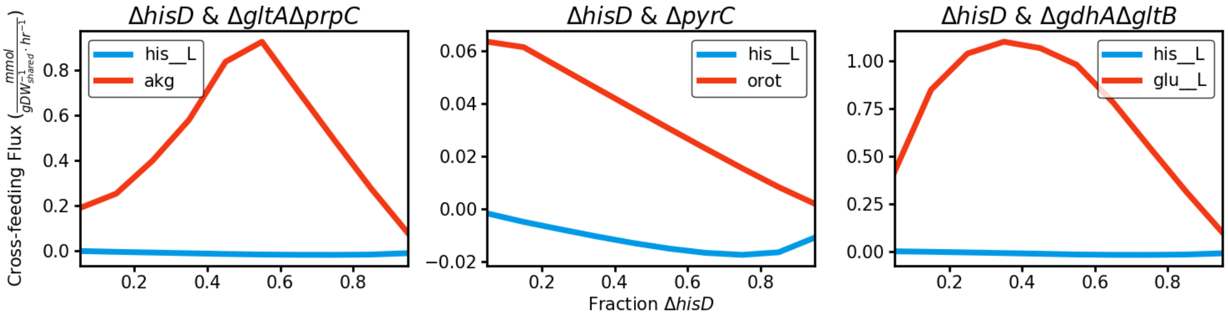


**Figure N. Optimal cross-feeding of ME-model simulation when limited to experimentally inferred metabolite.** The models were constrained to cross-feed only the metabolite that was inferred from the experimental resequencing data. (**Table 2**). Positive flux corresponds to cross-feeding from the Δ*hisD* strain to its partner and vice versa.

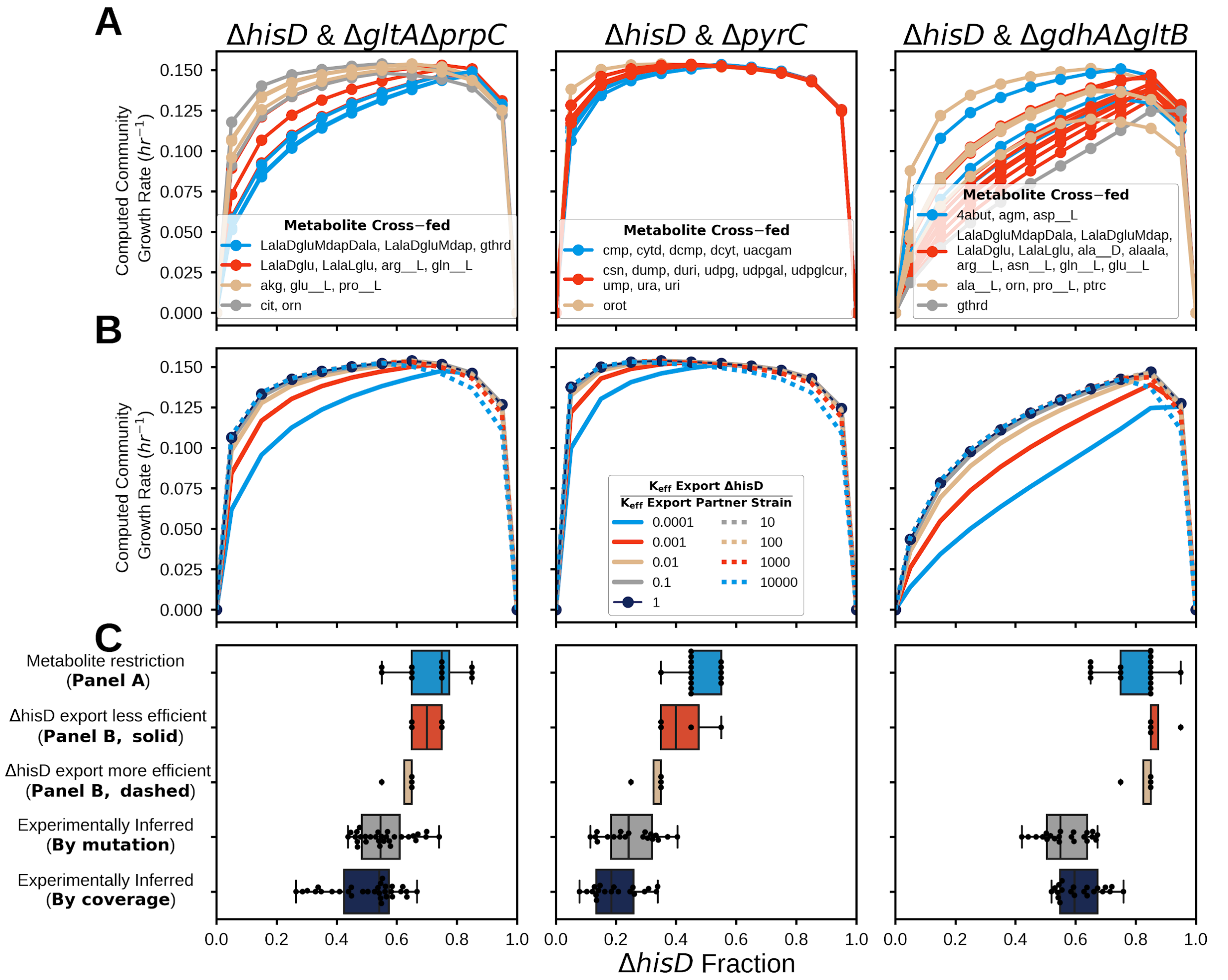


**Figure O. Community Modeling using *in vivo* estimated $k_{eff}$s.** Community ME-model predicted growth rates for fractional strain abundances of Δ*hisD* ranging from 0 to 1. (**A**) The effect of metabolite cross-feeding on community structure. Each curve was computed by allowing different metabolites to be cross-fed to the MSE strain. Similar curves were grouped by color. (**B**) The effect of varying the proteome efficiency of metabolite export on community structure (see **Methods**). The analysis was performed on models constrained to only cross-feed the metabolite that was inferred from the resequencing data (2-oxoglutarate, orotate, and L-glutamate, respectively) (**Table 2**). (**C**) Box plots of experimentally measured abundances for each sample (bottom two rows, gray, and dark blue) and the computationally-predicted optimal strain abundances following variation in the cross-feeding metabolite (top row, blue) and in strain proteome efficiency (second and third row, red, and yellow).

# References


1. Muffler A, Fischer D, Hengge-Aronis R. The RNA-binding protein HF-I, known as a host factor for phage Qbeta RNA replication, is essential for rpoS translation in Escherichia coli. Genes Dev. 1996;10: 1143–1151.
2. Wassarman KM, Repoila F, Rosenow C, Storz G, Gottesman S. Identification of novel small RNAs using comparative genomics and microarrays. Genes Dev. 2001;15: 1637–1651.
3. The UniProt Consortium. UniProt: the universal protein knowledgebase. Nucleic Acids Res. 2018; doi:10.1093/nar/gky092
4. Hwang E, Cheong H-K, Kim S-Y, Kwon O, Blain KY, Choe S, et al. Crystal structure of the EnvZ periplasmic domain with CHAPS. FEBS Lett. 2017;591: 1419–1428.
5. Eguchi Y, Okajima T, Tochio N, Inukai Y, Shimizu R, Ueda S, et al. Angucycline antibiotic waldiomycin recognizes common structural motif conserved in bacterial histidine kinases. J Antibiot . 2017;70: 251–258.
6. Seo SW, Gao Y, Kim D, Szubin R, Yang J, Cho B-K, et al. Revealing genome-scale transcriptional regulatory landscape of OmpR highlights its expanded regulatory roles under osmotic stress in Escherichia coli K-12 MG1655. Sci Rep. 2017;7: 2181.
7. Williams MD, Ouyang TX, Flickinger MC. Starvation-induced expression of SspA and SspB: the effects of a null mutation in sspA on Escherichia coli protein synthesis and survival during growth and prolonged starvation. Mol Microbiol. 1994;11: 1029–1043.
8. Hansen A-M, Lehnherr H, Wang X, Mobley V, Jin DJ. Escherichia coli SspA is a transcription activator for bacteriophage P1 late genes. Mol Microbiol. 2003;48: 1621–1631.
9. Hansen A-M, Qiu Y, Yeh N, Blattner FR, Durfee T, Jin DJ. SspA is required for acid resistance in stationary phase by downregulation of H-NS in Escherichia coli. Mol Microbiol. 2005;56: 719–734.
10. Hansen A-M, Qiu Y, Yeh N, Blattner FR, Durfee T, Jin DJ. SspA is required for acid resistance in stationary phase by downregulation of H-NS in Escherichia coli. Mol Microbiol. 2005;56: 719–734.
11. Urban JH, Vogel J. Translational control and target recognition by Escherichia coli small RNAs in vivo. Nucleic Acids Res. 2007;35: 1018–1037.
12. Muffler A, Fischer D, Hengge-Aronis R. The RNA-binding protein HF-I, known as a host factor for phage Qbeta RNA replication, is essential for rpoS translation in Escherichia coli. Genes Dev. 1996;10: 1143–1151.
13. Lelong C, Aguiluz K, Luche S, Kuhn L, Garin J, Rabilloud T, et al. The Crl-RpoS regulon of Escherichia coli. Mol Cell Proteomics. 2007;6: 648–659.
14. Ardeshir F, Ames GF. Cloning of the histidine transport genes from Salmonella typhimurium and characterization of an analogous transport system in Escherichia coli. J Supramol Struct. 1980;13: 117–130.
15. Baker KE, Ditullio KP, Neuhard J, Kelln RA. Utilization of orotate as a pyrimidine source by Salmonella typhimurium and Escherichia coli requires the dicarboxylate transport protein encoded by dctA. J Bacteriol. 1996;178: 7099–7105.
16. Sandegren L, Andersson DI. Bacterial gene amplification: implications for the evolution of antibiotic resistance. Nat Rev Microbiol. 2009;7: 578–588.
17. Pettersson ME, Sun S, Andersson DI, Berg OG. Evolution of new gene functions: simulation and analysis of the amplification model. Genetica. 2009;135: 309–324.

18. Feist AM, Zielinski DC, Orth JD, Schellenberger J, Herrgard MJ, Palsson BØ. Model-driven evaluation of the production potential for growth-coupled products of Escherichia coli. Metab Eng. 2010;12: 173–186.

19. Tepper N, Shlomi T. Predicting metabolic engineering knockout strategies for chemical production: accounting for competing pathways. Bioinformatics. 2010;26: 536–543.

20. Cronan JE Jr, Littel KJ, Jackowski S. Genetic and biochemical analyses of pantothenate biosynthesis in Escherichia coli and Salmonella typhimurium. J Bacteriol. 1982;149: 916–922.

21. Vander Horn PB, Backstrom AD, Stewart V, Begley TP. Structural genes for thiamine biosynthetic enzymes (thiCEFGH) in Escherichia coli K-12. J Bacteriol. 1993;175: 982–992.

22. Mee MT, Collins JJ, Church GM, Wang HH. Syntrophic exchange in synthetic microbial communities. Proc Natl Acad Sci U S A. 2014;111: E2149–56.

23. Somers JM, Amzallag A, Middleton RB. Genetic fine structure of the leucine operon of Escherichia coli K-12. J Bacteriol. 1973;113: 1268–1272.

24. Lee Y-J, Cho J-Y. Genetic manipulation of a primary metabolic pathway for L-ornithine production in Escherichia coli. Biotechnol Lett. 2006;28: 1849–1856.

25. Glansdorff N. TOPOGRAPHY OF COTRANSDUCIBLE ARGININE MUTATIONS IN ESCHERICHIA COLI K-12. Genetics. 1965;51: 167–179.

26. Bertels F, Merker H, Kost C. Design and characterization of auxotrophy-based amino acid biosensors. PLoS One. 2012;7: e41349.

27. Felton J, Michaelis S, Wright A. Mutations in two unlinked genes are required to produce asparagine auxotrophy in Escherichia coli. J Bacteriol. 1980;142: 221–228.

28. Pai CH. Biotin auxotrophs of Escherichia coli. Can J Microbiol. 1969;15: 21–26.
